# Route of Infection Strongly Impacts the Host-Pathogen Relationship

**DOI:** 10.3389/fimmu.2019.01589

**Published:** 2019-07-11

**Authors:** Aurore Demars, Aurore Lison, Arnaud Machelart, Margaux Van Vyve, Georges Potemberg, Jean-Marie Vanderwinden, Xavier De Bolle, Jean-Jacques Letesson, Eric Muraille

**Affiliations:** ^1^Unité de Recherche en Biologie des Microorganismes, Laboratoire d'Immunologie et de Microbiologie, NARILIS, Université de Namur, Namur, Belgium; ^2^Laboratory of Neurophysiology, Université Libre de Bruxelles, Brussels, Belgium; ^3^Laboratoire de Parasitologie, Faculté de Médecine, Université Libre de Bruxelles, Bruxelles, Belgium

**Keywords:** *Brucella melitensis*, brucellosis, infection control, live vaccine, virulence genes, reservoir cells

## Abstract

Live attenuated vaccines play a key role in the control of many human and animal pathogens. Their rational development is usually helped by identification of the reservoir of infection, the lymphoid subpopulations associated with protective immunity as well as the virulence genes involved in pathogen persistence. Here, we compared the course of *Brucella melitensis* infection in C57BL/6 mice infected via intraperitoneal (i.p.), intranasal (i.n.) and intradermal (i.d.) route and demonstrated that the route of infection strongly impacts all of these parameters. Following i.p. and i.n. infection, most infected cells observed in the spleen or lung were F4/80^+^ myeloid cells. In striking contrast, infected Ly6G^+^ neutrophils and CD140a^+^ fibroblasts were also observed in the skin after i.d. infection. The *virB* operon encoding for the type IV secretion system is considered essential to deflecting vacuolar trafficking in phagocytic cells and allows *Brucella* to multiply and persist. Unexpectedly, the Δ*virB Brucella* strain, which does not persist in the lung after i.n. infection, persists longer in skin tissues than the wild strain after i.d. infection. While the CD4^+^ T cell-mediated Th1 response is indispensable to controlling the *Brucella* challenge in the i.p. model, it is dispensable for the control of *Brucella* in the i.d. and i.n. models. Similarly, B cells are indispensable in the i.p. and i.d. models but dispensable in the i.n. model. γδ^+^ T cells appear able to compensate for the absence of αβ^+^ T cells in the i.d. model but not in the other models. Taken together, our results demonstrate the crucial importance of the route of infection for the host pathogen relationship.

## Introduction

Live attenuated vaccines (LAVs), composed of live pathogens that are made much less virulent than the pathogenic parental strains, are one of the most cost effective health tools in medical history [for review see (1–3)]. The advantages of LAVs include their mimicry of natural infections, the stimulation of long-term humoral and cellular immunity and their intrinsic adjuvant properties. First generation LAVs relied on empirical and somewhat unpredictable attenuation. In the present regulatory environment, the use of LAVs has been limited by safety concerns, especially due to the risk of reversion to wild-type virulence and the possibility of causing disease in immune compromised individuals. However, advances in immunology, molecular virology and bacteriology have paved the way for the rational design of LAVs while avoiding the unpredictability of empirical attenuation to thus reduce the safety risks.

Rational design of LAVs requires good knowledge of the *in vivo* infection process. In particular, the pathogen's cell cycle, main reservoirs of infection and the virulence genes involved in persistence of the pathogen can be characterized in order to help select the most effective candidate vaccines. However, one of the biggest remaining challenges is the identification of immune markers of protection, such as the type of T helper response and the lymphoid subpopulations associated with protective immunity (4). In the current study, we use the *Brucella* infection in mice as a model to investigate the impact of the route of infection on the identification of these parameters.

*Brucella* (an alphaproteobacterium) is a facultative intracellular Gram-negative coccobacillus that infects wild and domestic mammals and causes brucellosis. Human brucellosis is among the most common zoonoses (5). The vast majority of cases worldwide are attributed to *B. melitensis* [reviewed in Pappas et al. (6)]. Although it is rarely fatal, *Brucella* can cause a devastating multi-organ disease in humans with serious health complications in the absence of prolonged antibiotic treatment (6, 7). Human brucellosis primarily occurs following mucosal exposure to contaminated aerosols or ingestion of contaminated foods (8–12). However, in some occupational groups, such as cattle dealers, butchers, veterinarians, and farmers, it is well-documented that brucellosis can be acquired directly through contact of broken skin with infected animals (13, 14). The frequency of direct cutaneous infection is difficult to determine and could be underestimated as cutaneous manifestations of brucellosis are known to disappear spontaneously in patients (15).

During infection, *B. melitensis* mainly leads a stealthy intracellular lifestyle (16). Effector proteins secreted by the type IV secretion system (T4SS), which is encoded by the *virB* operon, are involved in the establishment of intracellular replicative niches. *B. melitensis* strains lacking a functional T4SS appear to be highly attenuated in mice and in their natural host, the goat [reviewed in De Jong and Tsolis (17)]. *In vitro* experiments using macrophage cell lines have shown that the T4SS is required for maturation of the *Brucella* phagosome into an endoplasmic reticulum-derived compartment (18).

Although the most frequent natural routes of *Brucella* infection are mucosal or cutaneous, the main experimental model for studying brucellosis in mice is intraperinoneal (i.p.) infection, which bypasses mucosal immune defenses and leads to infection that very rapidly becomes systemic. Over the last decade, our group characterized the phenotype of infected cells and the protective response against *Brucella* in an i.p. model (19, 20) and compared it to a model of intranasal (i.n.) infection (21). Taken together, those studies demonstrated that the lymphocyte populations required to control a *Brucella* challenge in each infectious model differed widely. The CD4^+^T cell-mediated Th1 response and B cells are indispensable in the i.p. model (20) but appear to be dispensable in the i.n. model (21).

To our knowledge, there is no described experimental model of cutaneous *Brucella* infection in mice. Only one study (22) describes cutaneous injection with *Brucella abortus* in the footpad of guinea pigs and the development of protective memory. Thus, in the present study, we developed and characterized an original intradermal (i.d.) infection model in mice. We compared the dissemination of wild-type and *virB*-deficient strains of *Brucella* and the type of cells infected and the protective immune response in the i.d., i.n., and i.p. models. This approach led us to conclude that the route of infection has unexpected major consequences on the host-pathogen relationship and should definitely be considered when selecting LAVs.

## Materials and Methods

### Ethics Statement

The procedures used in this study and the handling of the mice complied with current European legislation (Directive 86/609/EEC) and the corresponding Belgian law “Arrêté royal relatif à la protection des animaux d'expérience” of 6 April 2010 and published on 14 May 2010. The Animal Welfare Committee of the Université de Namur (UNamur, Belgium) reviewed and approved the complete protocol for *Brucella melitensis* infection (Permit Number: UN-LE-13/195).

### Mice and Bacterial Strains

Wild-type C57BL/6 and BALB/c mice were acquired from Harlan (Bicester, UK). TCR-δ^−/−^, CD3ε^−/−^, TCR-β^−/−^, MuMT^−/−^, CCR2^−/−^, and CCR7^−/−^ C57BL/6 were all purchased from The Jackson Laboratory (Bar Harbor, ME). IFN-γR^−/−^ (23) and IL-$12_{\text{p}35}^{-/-}$ C57BL/6 mice (24) were acquired from Dr. B. Ryffel (University of Orleans, France). IL17RA^−/−^ C57BL/6 mice (25) were acquired from Dr. K. Huygen (Belgian Scientific Institute for Public Health, Brussels, Belgium). TNFR1^−/−^ C57BL/6 (26) were acquired from Dr. C. De Trez (Vrije Universiteit Brussel). TAP1^−/−^ C57BL/6 mice (27) and MHCII^−/−^ C57BL/6 mice (28) were acquired from Jörg Reimann (University of Ulm, Ulm, Germany). All wild-type and deficient mice used in this study were bred in the animal facility of the Gosselies campus of the Université Libre de Bruxelles (ULB, Belgium). We used wild-type strains of *Brucella melitensis* 16M. We also used *Brucella melitensis* 16M stably expressing a rapidly maturing variant of the red fluorescent protein DsRed (29), the mCherry protein, under the control of the strong *Brucella* spp. promoter, p_sojA_. The construction of the mCherry-expressing *Brucella melitensis* (mCherry-*B*) strain has been described previously in detail (30). The Δ*virB* mutant was constructed in the mCherry-*B* strain by triparental mating to introduce the pJQ200 UC1-*virB* plasmid from the *E. coli* DH10B strain [described in Nijskens et al. (31)] into the mCherry-*B* strain using the *E. coli* MT 607 (pro-82 thi-I hsd R17 (r-m+) supE44 recA56 pRK600) strain [described in Casadaban and Cohen (32)], and the allelic replacement was performed as described previously for other gene deletions (33). Deletion of the *virB* operon was checked by PCR using the *virB*-F-check 5′-CGCTCGGCTATTATGACGGC-3′ and *virB*-R-check 5′-CGCCGATCATAACGACAACGG-3′ primers.

Cultures were grown overnight with shaking at 37°C in 2YT liquid medium (Luria-Bertani broth with double quantity of yeast extract) and were washed twice in RPMI 1640 (Gibco Laboratories; 3,500x g, 10 min) before inoculation of the mice.

*Brucella melitensis* was always handled under BSL-3 containment according to Council Directive 98/81/EC of 26 October 1998 and a law of the Walloon government of 4 July 2002.

### *Brucella melitensis* Staining With eFluor670

For some experiments, we stained *Brucella melitensis* with eFluor670 labeling. Cultures were grown overnight as indicated above and we then incubated the bacteria for 20 min in the dark with eFluor670 dye at the final concentration of 10 μM. After incubation, the bacteria were washed three times in PBS and once in RPMI before inoculation of the mice.

### Measurement of *Brucella melitensis* Multiplication *in vitro*

The growth of *Brucella melitensis* in liquid culture was monitored continuously using the multiwell Bioscreen system (Thermo Fisher, ref. 110001-536). The *B. melitensis* cultures in 2YT medium were centrifuged, washed once with PBS and diluted to an OD_600_ of 0.05 in 2YT to start culture in the Bioscreen system. Each sample (200 μl per well) was cultured at 37°C for 48 h.

### *Brucella melitensis* Infection *in vivo*

We used wild-type or mCherry-expressing *Brucella melitensis* in RPMI [described in Copin et al. (30)]. Control animals were inoculated with the same volume of PBS. The infectious doses were validated by plating serial dilutions of the inoculums. For i.n. infection, mice were anesthetized with a cocktail of Xylasine (9 mg/kg) and Ketamine (36 mg/kg) in PBS before being inoculated with 30 μl of the indicated dose of *Brucella melitensis*. For i.d. infection, mice were anesthetized by inhalation of Isofluran before injecting 20 μl of the indicated dose of *Brucella melitensis*. For i.p. infection, mice were injected with 500 μl intraperitoneally without anesthesia.

### Protocol for Secondary Infection With *Brucella melitensis*

C57BL/6 mice were immunized intranasally (i.n.), intradermally (i.d.) or intraperitoneally (i.p.) as indicated, with 2 × 10^4^ CFU of live wild type *B. melitensis*. The infectious doses were validated by plating serial dilutions of inoculums. 28 days after immunization, the mice were given antibiotics for 15 days to clear the infection. This oral treatment was a combination of rifampicin (12 mg/kg) and streptomycin (450 mg/kg; adapted from Vitry et al. (20)] prepared fresh daily and given in the drinking water. To ensure that the antibiotic treatment was effective, some mice in each group were sacrificed 1 week prior to the challenge and the CFU counts were evaluated in the spleen. After resting for an additional 15 days, the mice were challenged i.n., i.d. or i.p., as indicated with 2 × 10^4^ CFU of live mCherry-*B. melitensis* and sacrificed at the indicated times.

### Footpad Lesion Monitoring

The thickness of *Brucella melitensis* infected footpads was measured regularly with a metric caliper, and corresponded to the size of the footpad lesions.

### *Brucella melitensis* Counting in Mice

At the selected time post infection, we collected 75 μl of blood from the mice. They were then sacrificed by cervical dislocation. Immediately after sacrifice, the spleen, liver (one lobe), footpad, lung (left), mediastinal lymph node, thymus, muscle (1 square cm of thigh muscle), heart, brain, tail (2 mm), ovary, popliteal lymph node (depending on the experiment) were collected for bacterial counting. Tissues were crushed and transferred to PBS/0.1% X-100 Triton (Sigma-Aldrich). We performed successive serial dilutions in PBS to obtain the most accurate bacterial count and plated them on 2YT agar plates. The CFU were counted after 5 days of culture at 37°C.

### Cytofluorometric Analysis

The lungs were harvested and cut into small pieces. As described previously (21), spleens and footpad lesions were harvested, cut into small pieces and incubated for 1 h at 37°C with a mix of 100 μg/ml of DNAse I fraction IX (Sigma-Aldrich) and 1.6 mg/ml of collagenase (400 Mandl U/ml). The cells were then washed and filtered, and incubated with saturating doses of purified 2.4G2 (anti-mouse Fc receptor, ATCC) in 200 μl PBS, 0.2% BSA, 0.02% NaN3 (FACS buffer) for 20 min at 4°C to prevent antibody (Ab) binding to the Fc receptor.

3–5 × 10^6^ cells were stained on ice with various fluorescent mAb combinations in FACS buffer. We acquired the following mAbs from BD Biosciences: BV421-coupled T45-2342 (anti-F4/80), BV421-coupled M1/70 (anti-CD11b), fluorescein (FITC)-coupled 145-2C11 (anti-CD3ε), FITC-coupled 30-F11 (anti-CD45), FITC-coupled M1/70 (anti-CD11b), phycoerythrin (PE)-coupled HL3 (anti-CD11c), PE-coupled 1A8 (anti-Ly6G), allophycocyanin (APC)-coupled BM8 (anti-F4/80), biotin-coupled 2G9 (anti-MHCII, I-A/I-E). Biotin-coupled APA5 (anti-CD140a/ PDGFRA) was purchased from eBioscience. The biotin-coupled Abs were incubated with streptavidin-coupled APC for 30 min. The cells were analyzed on a BD FacsVerse flow cytometer. Dead cells and debris were eliminated from the analysis according to size and scatter.

### Fluorescence Microscopy of *Brucella melitensis in vitro*

*Brucella melitensis* strains were observed with a Nikon 80i (objective phase contrast ×100, plan Apo) connected to a Hamamatsu ORCA-ER camera. For the observation of *B. melitensis*, 2 μl of an exponential phase culture was dropped on an agarose pad (solution of 1% agarose in PBS) and sealed with VALAP (1/3 vaseline, 1/3 lanoline and 1/3 paraffin wax). Images were taken manually every 20 min at 32 °C with NIS-Element software (Nikon).

### Immunofluorescence Microscopy of Tissue

Spleens and lymph nodes were fixed for 2 h at RT in 2% paraformaldehyde (PFA) (pH 7.4), washed in PBS, and incubated overnight at 4°C in a 20% PBS-sucrose solution. Lungs were fixed for 20 min at RT in 2% PFA. Then, lungs were placed under a vacuum until no air was present in the lungs in 2% PFA for 2 h. After fixation, lungs were incubated overnight at 4°C in a 20% PBS-sucrose solution. Tissues were then embedded in Tissue-Tek OCT compound (Sakura), frozen in liquid nitrogen, and cryostat sections (5 μm) were prepared. For staining, tissue sections were rehydrated in PBS and incubated in a PBS solution containing 1% blocking reagent (Boeringer) (PBS-BR 1%) for 20 min before incubation overnight in PBS-BR 1% containing any of the following mAbs or reagents: DAPI nucleic acid stain Alexa Fluor 350, 488 phalloidin (Molecular Probes), APC-coupled BM8 (anti-F4/80, Abcam), Alexa Fluor 647-coupled M5/114.15.2 (anti-I-A/I-E, MHCII, BioLegend), Alexa Fluor 647-coupled HL3 (anti-CD11c, BD Biosciences), biotin-coupled 1A8 (anti-Ly6G, BioLegend), biotin-coupled APA5 (anti-CD140a/ PDGFRA, eBioscience). The biotin-coupled Ab was incubated with streptavidin-coupled APC for 2 h. Slides were mounted in Fluoro-Gel medium (Electron Microscopy Sciences, Hatfield, PA). Labeled tissue sections were visualized with an Axiovert M200 inverted microscope (Zeiss, Iena, Germany) equipped with a high-resolution monochrome camera (AxioCam HR, Zeiss). Images (1,384 × 1,036 pixels, 0.16 μm/pixel) were acquired sequentially for each fluorochrome with A-Plan 10x/0.25 N.A. and LD-Plan-NeoFluar 63x/0.75 N.A. dry objectives and recorded as eight-bit gray-level ^*^.zvi files. At least 3 slides were analyzed per organ from 3 different animals and the results are representative of 2 independent experiments.

### Confocal Microscopy

Confocal analyses were performed using a LSM780 confocal system fitted on an Observer Z 1 inverted microscope equipped with an alpha Plan Apochromat 63x/1.46 NA oil immersion objective (Zeiss, Iena, Germany). DAPI was excited using a 405 nm blue diode, and emission was detected using a band-pass filter (410–480 nm). The 488 nm excitation wavelength of the Argon/2 laser was used in combination with a band-pass emission filter (BP500-535 nm) to detect Alexa Fluor 488 phalloidin. The 543 nm excitation wavelength of the HeNe1 laser and a band-pass emission filter (BP580–640 nm) were used for the red fluorochrome mCherry. The 633 excitation wavelength of the HeNe2 laser and a band-pass emission filter (BP660–695 nm) were used for far-red fluorochromes such as APC. To ensure optimal separation of the fluorochromes, blue & red signals were acquired simultaneously in one track and green & far red signals were acquired in a second track. The electronic zoom factor and stack depth were adjusted to the region of interest while keeping image scaling constant (x-y: 0.066 micron, z: 0.287 micron). A line average of 4 was used and datasets were stored as 8-bit proprietary ^*^.czi files. The images were displayed using Zen2012 software (Zeiss) with linear manual contrast adjustment and exported as 8-bit uncompressed ^*^.TIF images. The figures, representing single optical sections across the region of interest, were prepared using the Canvas program.

### Enzyme-Linked Immunosorbent Assay (ELISA)

The presence of *Brucella melitensis* specific murine IgM, IgG1, IgG2a, and IgG3 was determined by ELISA. As already described (17), polystyrene plates (269620; Nunc) were coated with HK *B. melitensis* (10^7^ CFU/mL) and incubated overnight at 4°C. The plates were blocked for 2 h at RT with 200 μl/well of PBS-3.65% casein. Then, plates were incubated with 50 μl/well of plasma in serial dilutions in PBS-3.65% casein. The plasma of uninfected mice and PBS were used as negative controls and a 12B12 mAb specific to *Brucella* LPS (34) was used as a positive control. After four washes with PBS, isotype-specific goat anti-mouse HRP conjugated Ab were added (50 μl/well) at appropriated dilutions (anti-IgM from Sigma-Aldrich; LO-MG1-13 HRPO, LO-MG2a-9 HRPO, and LO-MG3-13 HRPO from LOIMEX). After 1 h of incubation at RT, plates were washed four times in PBS and 100 μl/well of substrate solution (BD OptEiA Kit) was added. After 15 min of incubation at RT in the dark, the enzyme reaction was stopped by adding 25 μl/well of 2 N H_2_SO_4_. The absorbance was measured at 450 nm.

### Statistical Analysis

We used a (Wilcoxon-)Mann-Whitney test provided by the GraphPad Prism software to statistically analyse our results. Each group of deficient mice was compared to the wild-type mice. We also compared each group with the other groups and displayed the results when required. Values of *p* < 0.05 were considered to represent a significant difference. ^*^, ^**^, ^***^ denote *p* < 0.05, *p* < 0.01, *p* < 0.001, respectively.

## Results

### Each Route of *Brucella* Infection Is Associated With a Specific Pattern of Organ Infection

In order to determine the impact of the route of *Brucella* infection on the pattern of infected organs in mice, we administered 2 × 10^4^ CFU of mCherry-*Brucella melitensis* via i.p., i.n. or i.d. route to wild-type C57BL/6 mice. Mice were bled for CFU counting in the blood and then sacrificed at 1 h, 1, 5, 12, 28, and 50 days post infection and a large number of organs and tissues were collected (lung and draining mediastinal lymph node (LN), spleen, liver, thymus, skeletal muscle of the thigh, heart, brain, fragment of the tail, ovary, footpad lesion, and draining popliteal LN) and the CFU count was measured by plating, as described in the Material and Methods.

**Figure 1:**
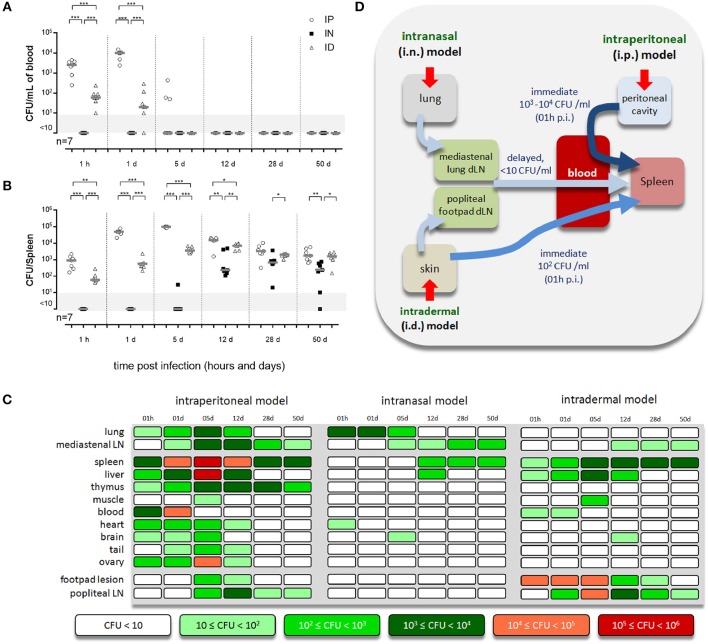
Each route of infection leads to a specific pattern of infected organs. Wild-type C57BL/6 mice were intraperitoneally (i.p.), intranasally (i.n.), or intradermally (i.d.) infected with a dose of 2 × 10^4^ CFU of mCherry-*B. melitensis* and sacrificed at the indicated times. **(A,B)** The data represent the CFU count per ml of blood or per spleen, as indicated. Gray bars represent the median. The significant differences between the indicated groups are marked with asterisks: ^*^p < 0.1, ^**^p < 0.01, ^***^p < 0.001. **(C)** The individual kinetic CFU data are summarized in a matrix giving the median level of CFU (*n* = 7) by tissue according to the route of infection. These results are representative of two independent experiments. h, hours; d, days; LN, lymph node; p.i., post infection; n, number of mice per group. **(D)** Hypothetical model summarizing the spread of *Brucella* from the primary site of infection to the spleen for each route of infection.

Our results, presented in Figures 1A,B and Figures S1, S2, showed drastic differences between the pattern of tissues infected via the i.p., i.n., and i.d. routes. A table providing an overview of the individual kinetic CFU data with the average CFU by tissue according to the route of infection is presented in Figure 1C. As previously reported by us, CFU are not detectable in blood following i.n. infection (21, 35) but are detectable following i.p. and i.d. infection (Figure 1A). However, CFU in blood were 10–100-fold lower following i.d. infection compared to i.p. infection. Despite these differences, which led to very different kinetics and levels of spleen infection in the first 12 days of infection, the spleen appears to be infected at almost comparable levels at 28 days post infection in all three models (Figure 1B). Several interesting observations can be made based on Figure 1C which summarizes our CFU data. They confirm that i.p. infection can be considered as a systemic model of infection as all tissues collected were found to be infected early and the majority of lymph nodes tested were still infected at 50 days post infection (Figure S3). In striking contrast, the i.n infection model produced a very narrow infection pattern: only the mediastinal LN (draining the lung) and spleen were infected persistently. The lung, liver, heart, and brain were infected significantly but only transiently. The i.d. infection model gave an intermediate infection pattern. The skin footpad lesion, popliteal LN (draining the footpad lesion), mediastinal LN, spleen, and liver were infected persistently. Skeletal muscle and the brain were only infected transiently. Importantly, we observed that *Brucella* persists over the long term, until 50 days post infection, mainly in lymphoid tissues, such as the LN and spleen, in all models and in the thymus in the i.p. model. A proposed model for the spread of *Brucella* from the primary site of infection to the spleen in the three models of infection is presented in Figure 1D.

### The Type of Cells Infected Depends on the Route of Infection

In the past (30, 36), we have used a *Brucella melitensis* 16M strain expressing the mCherry fluorescent tracer to detect and phenotype *Brucella* infected cells *in situ* by fluorescent microscopic analysis. However, this approach is very long and is not appropriate for the quantitative analysis of many organs. Flow cytometry analysis would be more suitable. Unfortunately, in the absence of an available yellow laser, the fluorescence of the mCherry-*Brucella* strain is too low to permit the direct detection of infected cells by flow cytometry. To avoid this technical problem, we developed a protocol using the dye eFluor^TM^ 670 (eFluor670) that produces intense fluorescent staining of the bacterial cell wall. Brief incubation of bacteria with eFluor670 leads to intense, stable and homogeneous staining of *Brucella* that can be detected by flow cytometry (Figure S4A. This staining does not negatively affect *Brucella* growth *in vitro* in a rich medium culture (Figure S4B) or in mice in the i.n (Figure S4C) and i.p. infection models (Figure S4D).

**Figure 2:**
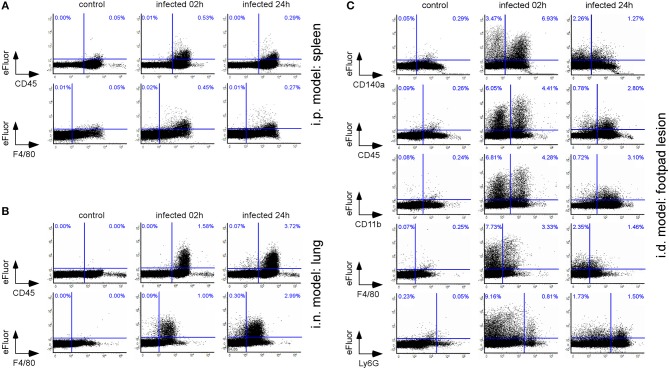
The type of cells infected is dependent on the route of infection. Wild-type C57BL/6 mice (*n* = 5) were infected intraperitoneally (i.p.) **(A)**, intranasally (i.n.) **(B)**, or intradermally (i.d.) **(C)** with a dose of 10^8^ **(A,B)** or 10^7^ **(C)** CFU of eFluor670 labeled *B. melitensis* and sacrificed at 2 and 24 h post infection. The spleen **(A)**, lung **(B)**, or footpad lesion **(C)** were harvested and the cells were isolated and then analyzed by flow cytometry for the expression of eFluor670, CD45, F4/80, CD140a, and CD11b as indicated. Numbers in blue indicate the percentage of eFluor670^+^ cells in each quadrant out of the total cells. These results are representative of three independent experiments.

**Figure 3:**
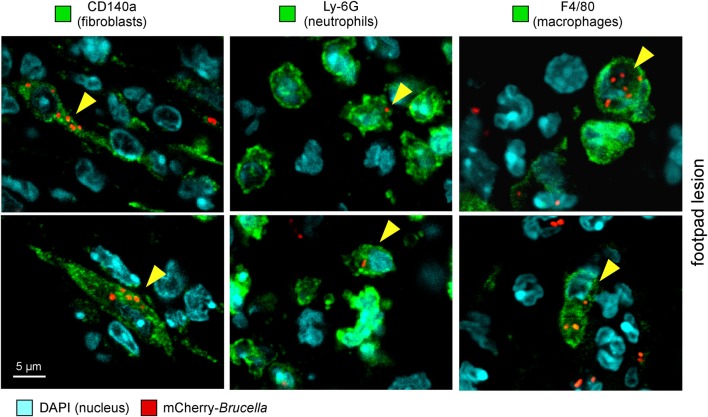
Following intradermal infection with *Brucella melitensis*, fibroblasts, neutrophils, and macrophages were found to be infected in the footpad lesion. Wild-type C57BL/6 mice were infected intradermally with a dose of 10^7^ CFU of mCherry-*B. melitensis*. Mice were sacrificed at 24 h post infection and the footpad lesions were collected and analyzed by confocal microscopy for the expression of mCherry and CD140a, LY6G, and F4/80 markers. The panels are color-coded with the text for DAPI, the antigen examined or mCherry-*Brucella*. Scale bar = 5 μm. Yellow arrowheads indicate the cells infected. The data are representative of two independent experiments.

As shown in Figure 2, eFluor670 staining allowed for rapid flow cytometric detection and quantification of *Brucella* infected cells present within tissues harvested at 2 and 24 h from infected mice. Our data confirm that, as previously reported by microscopic analysis, infected spleen cells in the i.p. model (30) (Figure 2A) and infected lung cells in the i.n. model (37) (Figure 2B) are mainly CD45^+^ F4/80^+^ myeloid cells, presumably red pulp macrophages and alveolar macrophages, respectively. In striking contrast, the same analysis of footpad lesion cells from the i.d. infection model showed that two clearly distinct cell populations, CD45^neg^ and CD45^+^, are infected by *Brucella* (37) (Figure 2C). Infected CD45^neg^ cells express high levels of the CD140a fibroblast marker and infected CD45^+^ cells express CD11b myeloid markers (Figure S5), which can be subdivided into CD11b^+^ F4/80^+^ Ly6G^neg/med^ and CD11b^+^ F4/80^neg/med^ Ly6G^high^ (Figure S6). Confocal microscopic analysis of footpad samples collected at 24 hours from infected mice demonstrated that CD140^+^, Ly6G^+^, and F4/80^+^ cells are indeed infected and display typical morphology of fibroblasts, neutrophils, and macrophages, respectively (Figure 3).

On the whole, these data showed that the type of cells infected by *Brucella* is strongly dependent on the route of infection. Although the infected cells are mainly macrophages in the i.p. and i.n. models, fibroblasts, and neutrophils are also infected in the i.d. model.

### Identification of Virulence Factors Is Affected by the Route of Infection and Organ Analyzed

In the rational development of LAVs, it is essential to identify virulence factors allowing for escape from the immune response and persistence in the host. LAVs must be able to establish infection in order to stimulate an adaptive immune response without persisting or disseminating deeply in the host. The most studied virulence factor in *Brucella* is undoubtedly the *virB* T4SS. *In vitro* in RAW 264.7 macrophages, a Δ*virB Brucella* strain is unable to escape the phagolysosome system and appears to be highly attenuated (38).

**Figure 4:**
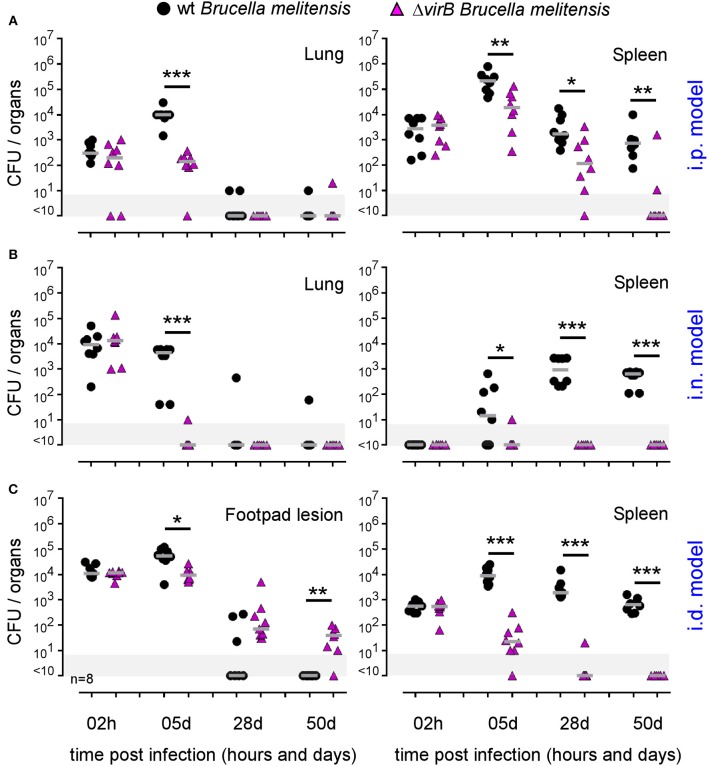
The essentiality of *virB* is dependent on the route of infection and organ. Wild-type C57BL/6 mice were infected intraperitoneally (i.p.) **(A)**, intranasally (i.n.) **(B)** or intradermally (i.d.) **(C)**, with a dose of 2 × 10^4^ CFU of mCherry-*B. melitensis* or with mCherry-Δ*virB B. melitensis*. Mice were sacrificed at the indicated times. The data represent the CFU count per lung, spleen, or footpad lesion. Gray bars represent the median. Significant differences between the indicated groups are marked with asterisks: ^*^p < 0.1, ^**^p < 0.01, ^***^p < 0.001. These results are representative of two independent experiments. h, hours; d, days; n, number.

In order to determine the impact of the route of infection on the type of virulence factors required to establish a successful infection *in vivo*, we compared the course of wild-type and Δ*virB Brucella* strains in wild-type C57BL/6 mice (Figure 4). As previously described (39), the Δ*virB Brucella* strain appears attenuated but able to persist for several weeks in the spleen in the i.p infection model (Figure 4A). In this model, we observed that the Δ*virB Brucella* strain is also attenuated in the lung. It persists at ~100 CFU between 2 h and 5 days post infection and is no longer detectable from 28 days. In striking contrast, in the i.n. model (Figure 4B), the Δ*virB Brucella* strain appears completely unable to multiply in the lung and reach the spleen. In the lung, the number of CFU drops by 3 log between 2 h and 5 days post infection. A complemented Δ*virB* strain recovers the ability to multiply in the lung, demonstrating that the inability of the Δ*virB* strain to persist in the lung is specific and related to the *virB* deficiency only (Figure S7). More surprising, in the i.d. model, the Δ*virB* strain persists longer in the primary footpad lesion than the wild-type *Brucella* strain. At 50 days post infection, the Δ*virB* strain is still present in the footpad while the wild-type strain has already disappeared at 28 days (Figure 4C). In the spleen, the Δ*virB* strain remains attenuated (Figure 4C).

**Figure 5:**
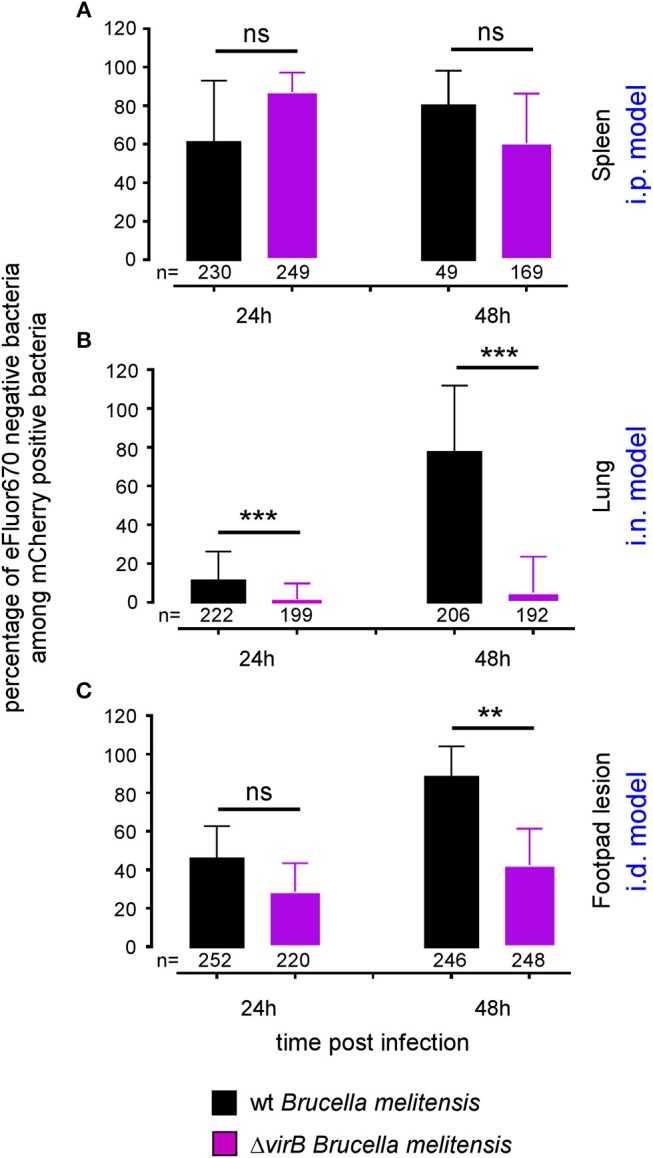
The Δ*virB B. melitensis* strain multiplies differently depending on the tissue. Wild-type C57BL/6 mice were infected intraperitoneally (i.p.) **(A)**, intranasally (i.n.) **(B)** or intradermally (i.d.) **(C)**, with a dose of 10^7^ CFU of eFluor670 labeled mCherry-*B. melitensis* or with eFluor670 labeled mCherry-Δ*virB B. melitensis*. Mice were sacrificed at the indicated times and the spleen, lung or footpad was harvested, fixed and analyzed by fluorescent microscopy. The data represent the percentage of eFluor670 negative bacteria among mCherry positive bacteria. Bars represent the standard deviation. Significant differences between the indicated groups are marked with asterisks: ^**^p < 0.01, ^***^p < 0.001. These results are representative of two independent experiments with 3 mice per condition. h, hours; n, number of bacteria analyzed in each condition.

**Figure 6:**
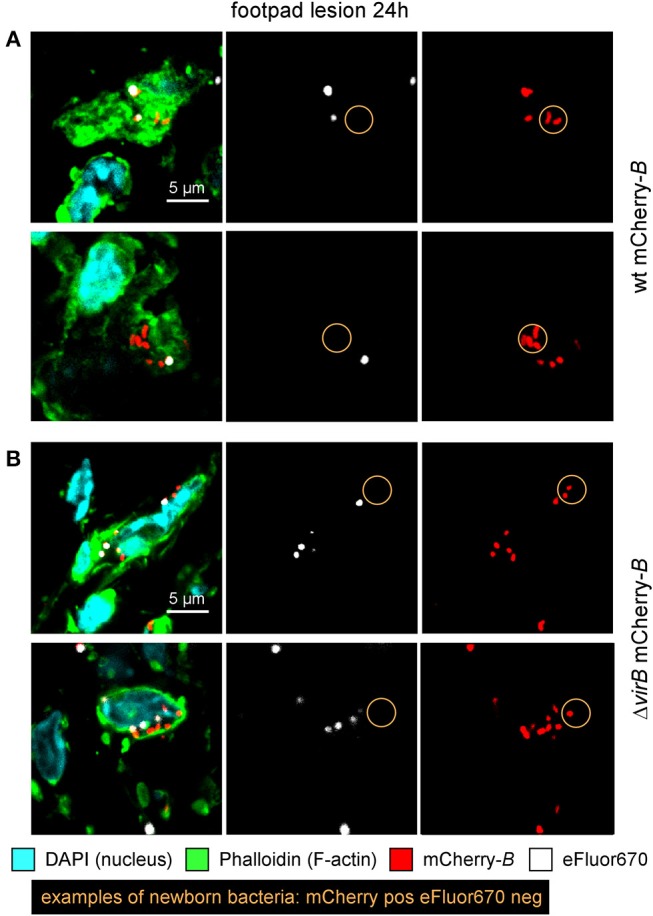
Δ*virB B. melitensis* actively multiplies in the footpad lesion. Wild-type C57BL/6 mice were infected intradermally (i.d.) with a dose of 10^7^ CFU of eFluor670 labeled wild-type or Δ*virB* mCherry-*B. melitensis*. Mice were sacrificed at 24 h post infection and the footpad was harvested, fixed, and analyzed by confocal microscopy. **(A,B)** show representative images for wild-type or Δ*virB* mCherry-*B. melitensis*, respectively. The panels are color-coded with the text for DAPI, Phalloidin, mCherry, and eFluor670. Scale bar = 5 μm. pos, positive; neg, negative.

The differing behavior of the Δ*virB* strain observed depending on the route of infection and the tissue may be due to a difference in the ability to actively multiply or to passively persist (i.e., persistence without multiplication). *Brucella* has been widely described to display atypical unipolar growth (40). Unlabelled daughter cells can be visualized upon the resumption of growth following staining of the bacteria with Texas red conjugated to succinimidyl ester (TRSE) (41). Here, we chose to replace TRSE with eFluor670 staining, which is compatible with the mCherry *Brucella* strain. Thus, newly formed bacteria, called “newborn” bacteria, appear as mCherry^+^ eFluor670^neg^ (Figure S8A). We confirmed by fluorescence microscopy the stability and absence of transfer of eFluor670 staining during *Brucella* growth and division *in vitro* (Figure S8B). Using this tool, we tried to quantify multiplication of the wild-type and Δ*virB Brucella* strains *in situ*. Mice were infected by i.p., i.n. or i.d. route with eFluor670-stained wild-type mCherry-*B* and Δv*irB* mCherry-expressing *Brucella* strains. 24 and 48 h later, mice were sacrificed and the organs of interest were harvested and analyzed by confocal microscopy. Figures 5A–C show the frequencies of mCherry^+^ eFluor670^neg^ (newborn) bacteria among the mCherry^+^ bacteria in the spleen, lung and footpad lesion at 24 and 48 h post infection from i.p., i.n., or i.d. infected mice, respectively. Figure 6 presents representative images from infected footpads. As expected, wild-type *Brucella* appears able to multiply in all tissues analyzed based on the abundant presence of mCherry^+^ eFluor670^neg^ newborn bacteria at 48 h post infection. The frequency of newborn *Brucella* in the spleen from wild-type and Δ*virB Brucella* infected mice was similar (Figure 5A). In contrast, the frequency of newborn *Brucella* in the lung from Δ*virB Brucella* infected mice is strongly reduced (~15 times) compared to wild-type *Brucella* infected mice (Figure 5B). In the footpad lesion, the situation appears to be intermediate. The frequency of newborn *Brucella* from Δ*virB Brucella* infected mice is only reduced by ~2-fold. These results suggest that the absence of persistence of Δ*virB Brucella* in the lung is due to a reduced ability to multiply and that persistence in the spleen and footpad lesion is associated with active multiplication.

We can conclude that the impact of *virB* deficiency appears vary widely as a function of the route of infection and organ analyzed, suggesting that the identification of virulence factors could be strongly impacted by these parameters.

In the next part of this article, we will focus on the impact of the route of infection on the type of immune response and lymphocyte populations involved in primary and secondary control of *Brucella melitensis* infection.

### Th1 Immune Response Is Crucial to Control of the Primary Cutaneous *Brucella* Infection

**Figure 7:**
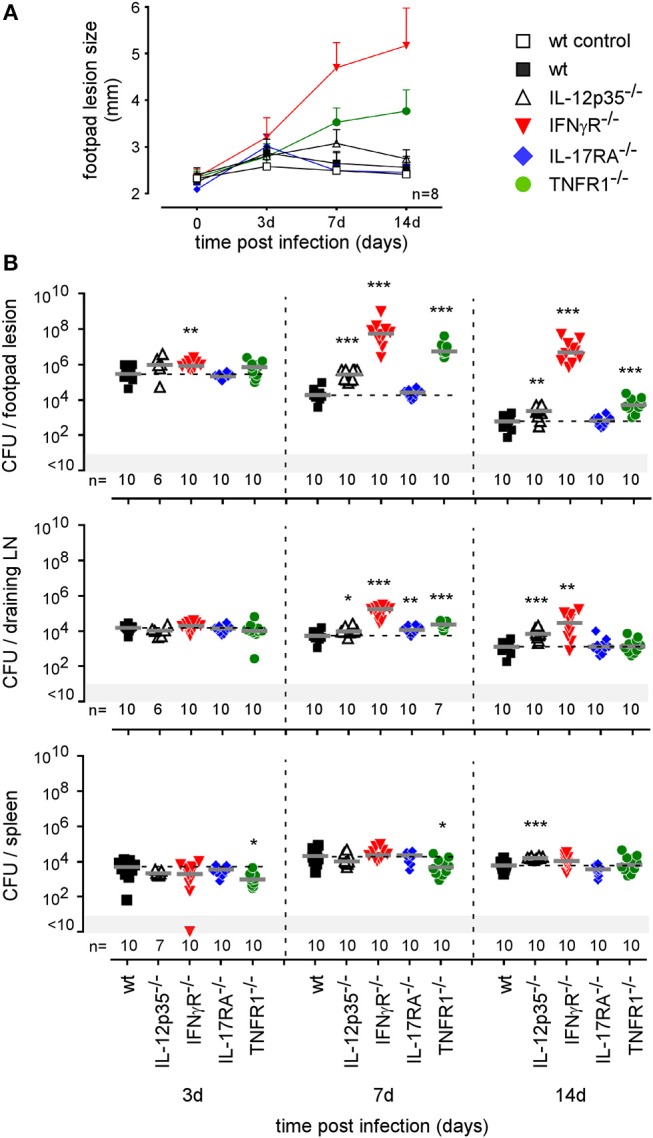
Intradermally infected IFNγR and TNFR1^−/−^ mice display significant footpad swelling and CFU counts. Wild-type, IL12p35^−/−^, IFNγR^−/−^, IL-17RA^−/−^, and TNFR1^−/−^ C57BL/6 mice were infected intradermally with a dose of 2 × 10^4^ CFU of mCherry-*B. melitensis*. **(A)** At the indicated time post infection, the swelling of the lesion was measured for each mouse. The error bars are the SD. **(B)** At the indicated times, mice were sacrificed and the footpad, draining popliteal lymph node, and spleen were harvested. The data represent the CFU count per organ. Gray bars represent the median. The significant differences between the indicated groups are marked with asterisks: ^**^p < 0.01, ^***^p < 0.001. These data are representative of two different experiments. d, days; n, number; LN, lymph node.

To our knowledge, the type of immune mechanism underlying cutaneous *Brucella* infection has never been investigated in a mouse model. As reported in i.p. (42) and i.n. (21) infectious models, BALB/c mice appear to be more susceptible to cutaneous *B. melitensis* infection compared to C57BL/6 mice (Figure S9). In order to identify the T helper subset of immune response associated with *Brucella* control in skin lesions, we compared the course of *Brucella melitensis* infection in wild-type, IL-$12_{\text{p}35}^{-/-}$, IFNγR^−/−^, IL-17RA^−/−^, and TNFR1^−/−^ C57BL/6 mice. We observed that *Brucella* infection in wild-type and IL-17RA^−/−^ mice leads to moderate footpad lesions at 3 days post infection that disappeared spontaneously at 7 days (Figure 7A). The lesions persisted longer in IL-$12_{\text{p}35}^{-/-}$ mice, but regressed spontaneously at 14 days. In striking contrast, lesions in IFNγR^−/−^ and TNFR1^−/−^ mice displayed uncontrolled growth until 14 days (Figure 7A) and become necrotic (data not shown). Growth and necrosis of the lesion were higher in IFNγR^−/−^ mice. The severity of the footpad lesion in all groups of mice appeared to be closely correlated with the corresponding CFU level (Figure 7B). We have previously reported (21) a dramatic difference between IL-$12_{\text{p}35}^{-/-}$ and IFNγR^−/−^ mice in control of a *Brucella* infection in the i.n. infection model. In this model, IFNγR^−/−^ mice, but not IL-$12_{\text{p}35}^{-/-}$ mice, display severe neutrophilia and succumb to the infection, suggesting that this lack of control and mortality result from the complete absence of IFN-γ and are not proportional to its level.

**Figure 8:**
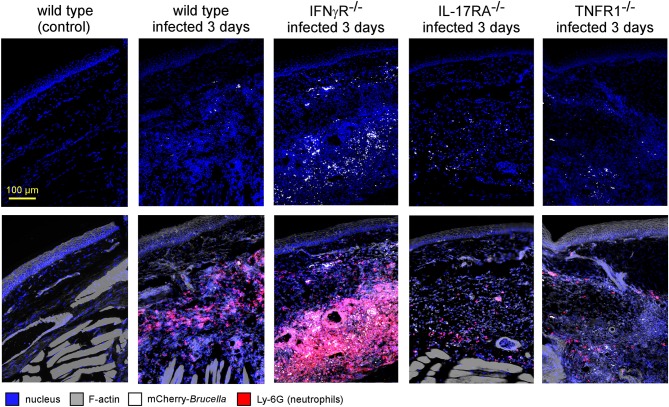
IFNγR deficiency leads to the massive recruitment of neutrophils in dermal tissue. Wild-type, IFNγR^−/−^, IL-17RA^−/−^, and TNFR1^−/−^ C57BL/6 mice were infected intradermally with a dose of 2 × 10^4^ CFU of mCherry-*B. melitensis*. Control wild-type mice were injected with PBS. Mice were sacrificed at 3 days post infection and the footpad was harvested and analyzed by fluorescent microscopy. The panels are color-coded with the text for DAPI, phalloidin, mCherry-*B.melitensis*, and LY-6G. Scale bar = 100 μm. These data are representative of two different experiments.

Fluorescent microscopic analysis of the footpad lesion at 3 days in wild-type, IL-17RA^−/−^ and TNFR1^−/−^ infected mice showed a moderate presence of neutrophils, identified by Ly-6G staining (Figure 8). In contrast, the dermis of the lesion from infected IFNγR^−/−^ mice displayed very intense neutrophil recruitment. This was confirmed by flow cytometric analysis of the footpad lesion (Figure S10A). In wild-type mice, *Brucella* infection led to the recruitment of CD11b^+^ F4/80^+^ Ly6G^neg^ monocytes that peaked at 7 days post infection with only moderate recruitment of CD11b^+^ F4/80^neg^ Ly6G^+^ neutrophils. In infected IFNγR^−/−^ mice, neutrophils constituted the major population in the footpad lesion and the frequency of monocytes appeared to be strongly reduced (Figure S10B).

**Figure 9:**
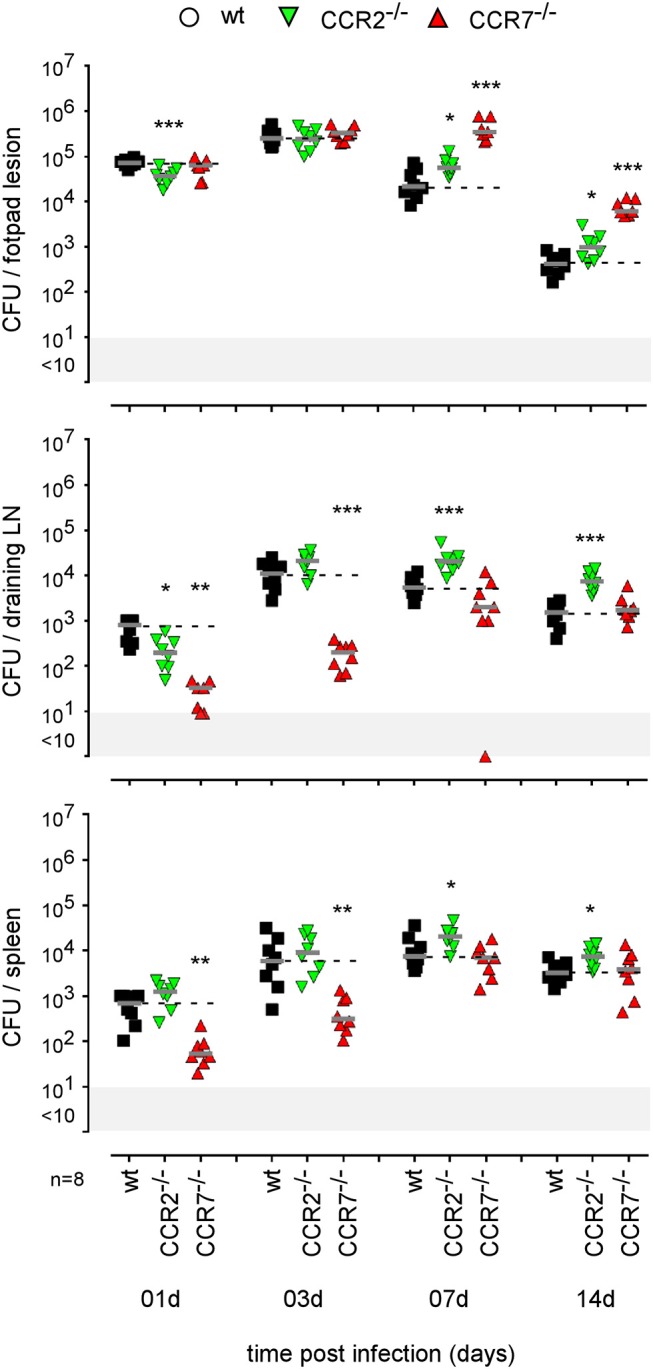
Impact of CCR2 and CCR7 deficiency on the course of intradermal *Brucella* infection. Wild-type, CCR2^−/−^, and CCR7^−/−^ C57BL/6 mice were infected intradermally with a dose of 2 × 10^4^ CFU of *B. melitensis* and sacrificed at the indicated times. The data represent the CFU count per organ. Gray bars represent the median. The significant differences between the indicated groups are marked with asterisks: ^*^p < 0.1, ^**^p < 0.01, ^***^p < 0.001. These results are representative of three independent experiments. LN, lymph node; d, days; n, number.

**Figure 10:**
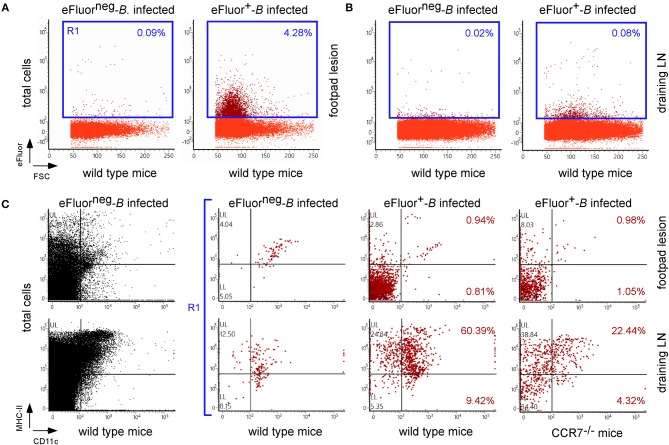
CCR7 deficiency reduced the frequency of infected dendritic cells in popliteal draining lymph nodes. Wild-type and CCR7^−/−^ C57BL/6 mice were infected intradermally with a dose of 10^7^ CFU of eFluor670^+^ labeled *B. melitensis* and sacrificed at 24 h post infection. The footpad lesion and the popliteal draining lymph nodes were harvested and the cells were isolated and then analyzed by flow cytometry for the expression of eFluor670, MHCII, and CD11c as indicated. The data represent the frequency of eFluor670^+^ cells in cells from footpad lesions **(A)** and popliteal draining lymph nodes **(B)** and the phenotype of eFluor670^+^ cells in these tissues **(C)**. Percentage indicates the percentage of cells in each quadrant out of the total cells **(A,B)** or of the eFluor670^+^ cells **(C)**. LN, lymph node. These results are representative of three independent experiments.

As our microscopic analysis showed that F4/80^+^ monocytes/macrophages appear to be infected by *Brucella* in the skin of i.d. infected mice (Figure 3) and their recruitment is correlated with the control of *Brucella* (Figure S10B), we investigated the impact of chemokine receptor CCR2 and CCR7 deficiency on *Brucella* control in our i.d. infection model. Monocyte emigration from bone marrow during bacterial infection requires signals mediated by CCR2 (43) but seems to be dispensable for the maintenance of dermal macrophages and dendritic cells (44). In contrast, CCR7 regulates the migration of inflammatory monocytes (45), and dendritic cells (46) from skin to the draining lymph node under inflammatory conditions. We observed that i.d. infected CCR2^−/−^ mice display delayed monocyte recruitment and a higher rate of neutrophils at 7 days post infection in the footpad lesion (Figure S10B) associated with an increased CFU count in the footpad lesion, popliteal draining LNs, and spleen (Figure 9), confirming the importance of monocyte recruitment in the local and systemic control of *Brucella* infection. Interestingly, CCR7 deficiency led to retention of *Brucella* in the lesions and seemed to delay its dissemination to the popliteal draining LNs and spleen, suggesting that *Brucella* dissemination is partially dependent on monocyte and dendritic cell migration. In agreement, flow cytometry analysis of footpad lesions and popliteal draining LNs from mice i.d. infected with eFluor670^+^ bacteria showed that, while the eFluor670^+^ cells in the lesion are mainly CD11c and MHCII negative, they are mostly positive in the LNs (Figures 10A–C). Interestingly, the frequency of CD11c^+^ MHCII^+^ infected dendritic cells is drastically reduced in CCR7^−/−^ mice compared to wild-type mice (Figure 10C).

**Figure 11:**
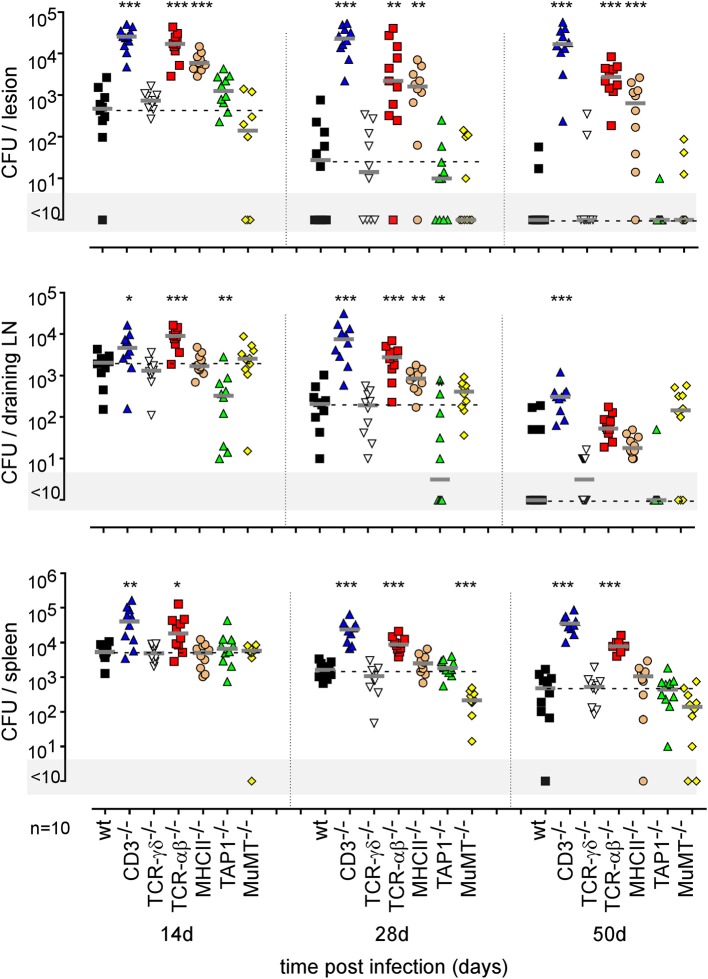
Impact of various lymphocytes deficiencies on the course of intradermal *Brucella* infection. Wild-type, CD3^−/−^, TCRαβ^−/−^, TCRγδ^−/−^, MHCII^−/−^, TAP1^−/−^, and MuMT^−/−^ C57BL/6 mice were infected intradermally with a dose of 2 × 10^4^ CFU of *B. melitensis* and sacrificed at the indicated times. The data represent the CFU count per organ. Gray bars represent the median. The significant differences between the indicated groups are marked with asterisks: ^*^p < 0.1, ^**^p < 0.01, ^***^p < 0.001. These results are representative of three independent experiments. LN, lymph node; d, days; n, number.

Finally, using a battery of genetically-deficient C57BL/6 mice, we investigated the role of lymphoid populations in the control of i.d. *Brucella melitensis* infection. Comparison of the course of *Brucella* infection in wild-type, CD3^−/−^ (deficient for T cells), TCR-δ^−/−^ (deficient for γδT cells), TCR-β^−/−^ (deficient for both CD4^+^T and CD8^+^T cells), MHCII^−/−^ (deficient for CD4^+^T cells), TAP1^−/−^ (deficient for CD8^+^Tcells), and MuMT^−/−^ (deficient for B cells) mice showed that CD4^+^T cells are strictly required for *Brucella* control in the footpad lesion and popliteal draining lymph node (Figure 11). Interestingly, CD4^+^T cells appear to be dispensable in the spleen, as only CD3 and TCR-β deficiency negatively impaired *Brucella* control there. CD8^+^T cell deficiency significantly favored *Brucella* control in the draining lymph node but not in the lesion or spleen. This may be the consequence of the higher number of CD4^+^T cells in absence of CD8^+^T cell in TAP1^−/−^ mice. In a previous study (47), we demonstrated that even though the amounts of IFN-γ produced by CD8^+^T cells and CD4^+^T cells are similar, CD8^+^T cells are unable to replace CD4^+^T cells in their control of *Brucella* infection, suggesting that CD4^+^T cells deploy as yet unidentified effector mechanisms that may be independent of IFN-γ. In the i.d. infection model, γδ^+^T cells appeared to be dispensable for the control of i.d. *Brucella* infection. Despite the detectable humoral response (Figure S11A), similar to that observed in the i.p. infection model (Figure S11B), B cells also appeared to be dispensable. However, though the finding was not statistically significant, we did observe a repeated tendency of MuMT mice to display increased CFU counts in draining lymph nodes at 50 days post infection (Figure 11).

Taken together, our data demonstrate that the control of primary cutaneous *Brucella* infection mainly required an IFN-γ-mediated Th1 response, TNF-α production and CD4^+^ T cells but not an IL-17RA-mediated Th17 response. They also show that monocyte recruitment plays a crucial role in both the control and dissemination of cutaneous *Brucella* infection.

### The Type of Immune Response Controlling Secondary *Brucella* Infection Is Dependent on the Route of Infection

In order to establish the main features of the protective immune response induced by i.d. *Brucella* infection, we started by comparing the course of i.d. mCherry-*B. melitensis* infection in naive mice (primary infection group) and in mice previously i.d. infected with wild-type-*B. melitensis* for 28 days and then treated with antibiotics (secondary infection group), as described in the Material and Methods.

Antibiotic treatment is indispensable to the comparison of wild type and genetically deficient mice. It is well known that the persistence of a pathogen generates chronic inflammation and can cause a “Mackaness effect”(48, 49). Without antibiotics, the level of persistence of the vaccine strain would be very different between wild-type mice and mice deficient for key components of the immune response against *Brucella*, which would make our results very difficult to interpret. It has been reported that Rev1 infection in sheep (50, 51) and rams (52) is fully cleared between the second and third month after subcutaneous or conjunctival vaccination. Thus, by eliminating the primary infection at 28 days in our experimental model, we are not so far away from the conditions of the natural host vaccinated with the reference Rev1 vaccine against *Brucella melitensis*.

**Figure 12:**
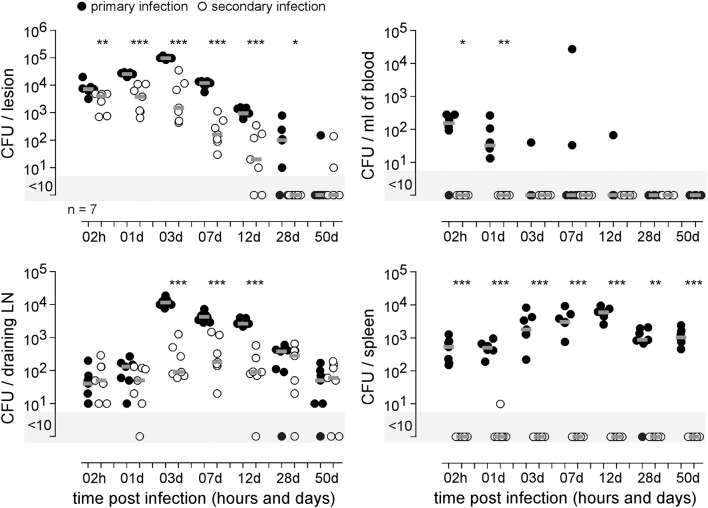
Comparison of protection in control mice and mice previously immunized with live *B. melitensis*. C57BL/6 mice were immunized intradermally (i.d.) with 2 × 10^4^ CFU of live wild type *B. melitensis* and treated with antibiotics, as described in the Materials and Methods. Naive (primary infection group) and immunized (secondary infection group) mice were challenged i.d. with 2 × 10^4^ CFU of live mCherry-*B. melitensis* and sacrificed at the indicated times. The data represent the CFU count per organ or per ml of blood. Gray bars represent the median. Significant differences between the indicated groups are marked with asterisks: ^*^p < 0.1, ^**^p < 0.01, ^***^p < 0.001. These results are representative of three independent experiments. LN, lymph node; d, days; h, hours; n, number.

Our results (Figure 12) demonstrated that i.d. infection leads to the development of an adaptive immune response able to efficiently control secondary i.d. infection in the footpad lesion, blood, popliteal draining lymph node and spleen. It is interesting to note that while *Brucella* is almost completely eliminated in the footpad lesion, blood, and spleen, it nevertheless persists at a significant level in the lymph nodes, suggesting that it may be sheltered from the adaptive immune response at this site.

**Figure 13:**
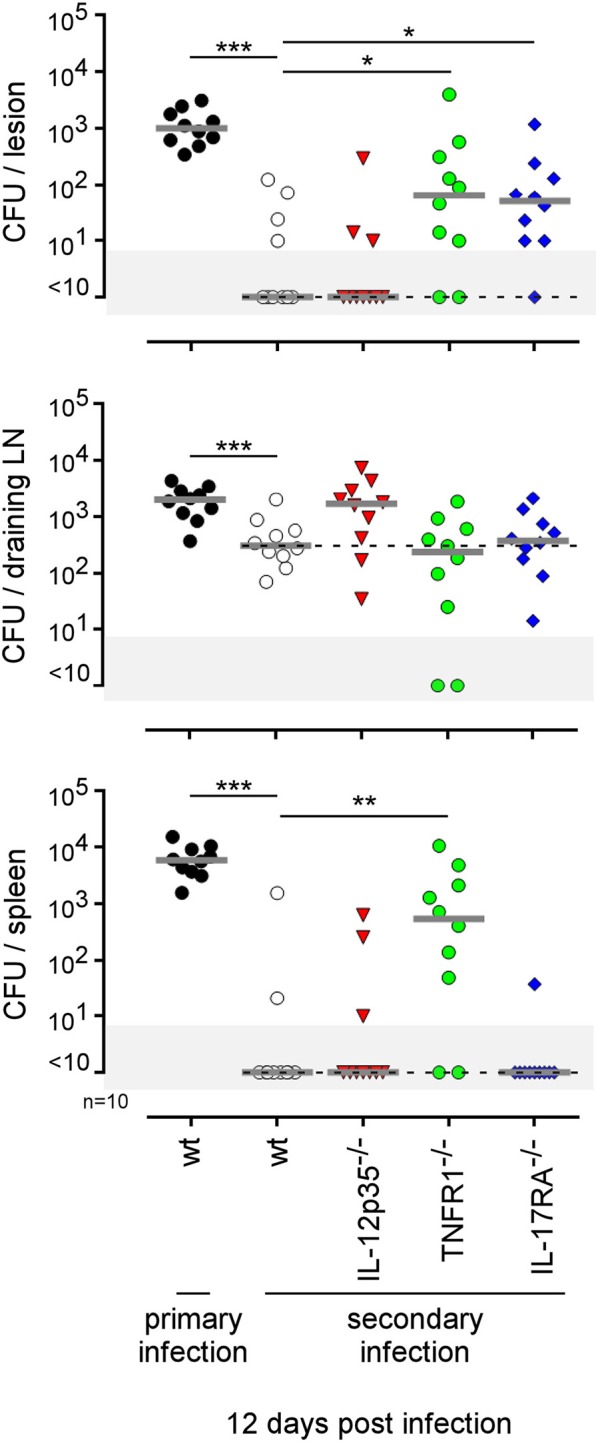
Comparison of protection in wild-type and various deficient mice previously immunized by intradermal route with live *B. melitensis*. Wild-type, IL-12p35^−/−^, TNFR1^−/−^, IL-17RA^−/−^ C57BL/6 mice were immunized intradermally (i.d.) with 2 × 10^4^ CFU of live wild-type *B. melitensis* and treated with antibiotics, as described in the Materials and Methods. Naive (primary infection group) and immunized (secondary infection group) mice were challenged i.d. with 2 × 10^4^ CFU of live mCherry-*B. melitensis* and sacrificed at 12 days post infection. The data represent the CFU count per organ. Gray bars represent the median. The significant differences between the indicated groups are marked with asterisks: ^*^p < 0.1, ^**^p < 0.01. These results are representative of three independent experiments. LN, lymph node; n, number.

**Figure 14:**
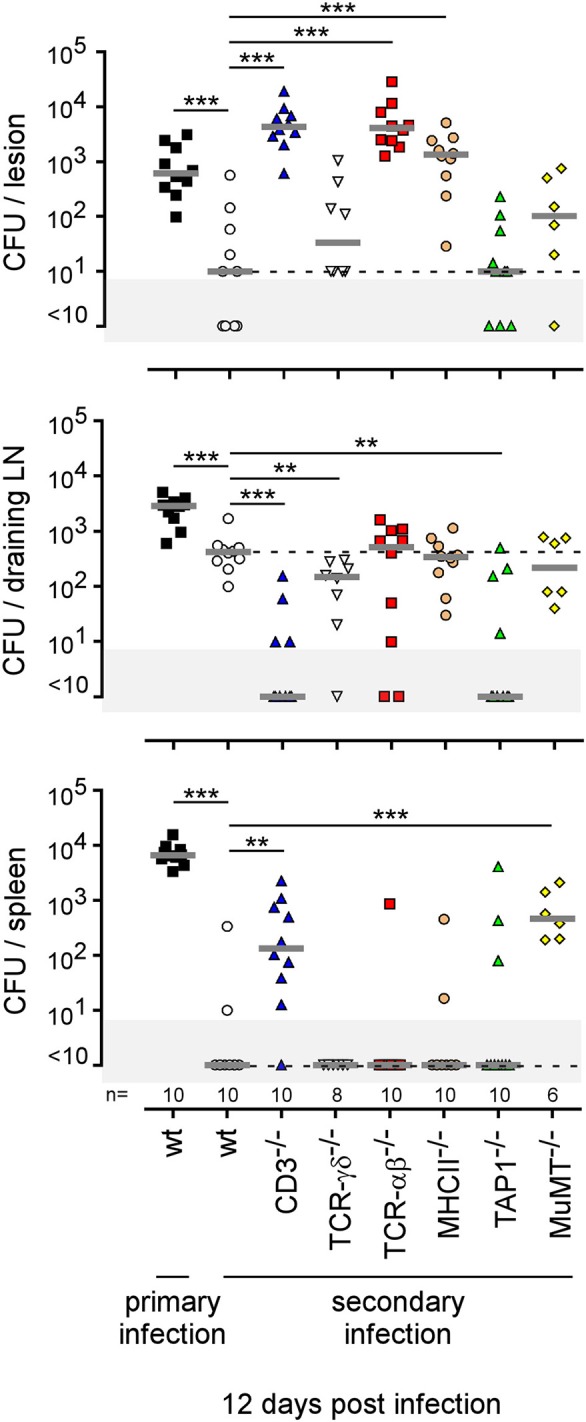
Comparison of protection in wild-type and various lymphocyte-deficient mice previously immunized by intradermal route with live *B. melitensis*. Wild-type, CD3^−/−^, TCRαβ^−/−^, TCRγδ^−/−^, MHCII^−/−^, TAP1^−/−^, and MuMT^−/−^ C57BL/6 mice were immunized intradermally (i.d.) with 2 × 10^4^ CFU of live wild-type *B. melitensis* and treated with antibiotics, as described in the Materials and Methods. Naive (primary infection group) and immunized (secondary infection group) mice were challenged i.d. with 2 × 10^4^ CFU of live mCherry-*B. melitensis* and sacrificed at 12 days post infection. The data represent the CFU count per organ. Gray bars represent the median. The significant differences between the indicated groups are marked with asterisks: ^**^p < 0.01, ^***^p < 0.001. These results are representative of three independent experiments. LN, lymph node; n, number.

The type of immune mechanism required for the protective secondary immune response appears to be dramatically dependent on the tissue analyzed. As shown in genetically-deficient mice, effective control in the footpad lesion is dependent on TNFR1 and IL-17RA but not IL-12_p35_ and, in the spleen, is dependent on TNFR1 but not IL-17RA and IL-12_p35_. In the popliteal lymph node, the absence of IL-12_p35_, IL-17RA, or TNFR1 does not have a significant impact on the CFU count (Figure 13). Similarly, only CD4^+^T cells, and no other lymphoid populations, are essential to control *Brucella* in the footpad lesion, but CD4^+^T cells are dispensable in the popliteal draining lymph node and spleen. In the spleen, B cells are strictly required but only the absence of all T cells in CD3^−/−^ mice leads to a significant loss of *Brucella* control, suggesting that the absence of a T cell subpopulation can be compensated for by the other subpopulations in this organ. Unexpectedly, the absence of T cells in CD3^−/−^ mice, and especially of CD8^+^T cells in TAP1^−/−^ mice, improved the control of *Brucella* in popliteal draining lymph nodes, suggesting that *Brucella* persistence there implicates CD8^+^T cells (Figure 14). Regulatory CD8^+^CD122^+^PD-1^+^T cells, which are located in lymph nodes and act in a non-antigen-specific manner, have been described in persistent viral infection (53, 54). These cells have not been described in a *Brucella* model but could be responsible for this phenomenon.

It would also have been interesting to test the ability of IFN-γR^−/−^ mice to develop a protective memory response against *Brucella*. However, these mice develop highly necrotic footpad lesions and, for ethical reasons, we therefore limited our observations of these mice to 14 days post infection.

**Figure 15:**
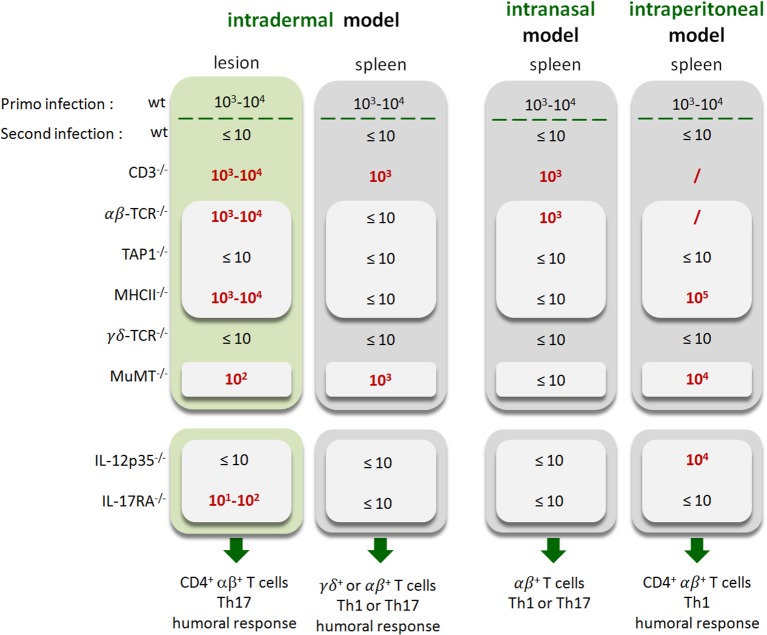
Summary of the immune response components indispensable to the control of secondary *Brucella* infection as a function of the route of infection. Wild-type, CD3^−/−^, TCRαβ^−/−^, TCRγδ^−/−^, MHCII^−/−^, TAP1^−/−^, MuMT^−/−^, IL-12p35^−/−^, and IL-17RA^−/−^ C57BL/6 mice were infected intradermally (i.d.), intranasally (i.n.) or intraperitoneally (i.p.) with 2 × 10^4^ CFU of live wild-type *B. melitensis* and treated with antibiotics, as described in the Materials and Methods. Naive (primary infection group) and immunized (second infection group) mice were challenged i.d., i.n., or i.p. with 2 × 10^4^ CFU of live mCherry-*B. melitensis* and sacrificed at 12 days post infection (for i.d.) or 28 days post infection (for i.n. and i.p.). The data represent a qualitative approximation of the CFU count per organ in the present article (i.d. model and Figure S8 for i.p. model) or in our group's previous article characterizing the i.p. (20) and i.n. (21) models. The CFU counts of mice which do not control the secondary infection are in red. “/”, not tested. Note that “Th1 or Th17” indicates that the Th1 response can compensate for the absence of the Th17 response and vice versa.

Lastly, to summarize the impact of the route of infection on the type of protective immune memory response, we compared the results obtained in the i.d. infection model with those obtained by our group in the i.p. (20) and i.n. (21) infection models (Figure 15). Note that the requirements were completed in the i.p. model to allow for a full comparison with other models (Figure S12). Figure 15 shows clearly that the type of lymphoid cells and T helper response required to control secondary *Brucella* infection is strongly dependent on the route of infection and the tissues infected. For example, CD4^+^T cells are indispensable in the footpad lesion in the i.d. model and in the spleen in the i.p. model but are dispensable in the spleen level in both the i.d. and i.p. models. B cells are indispensable in the i.d. and i.p. models but dispensable in the i.n. model. The IL-12_p35_ dependent Th1 response is only required in the spleen in the i.p. model and in the footpad lesion in the i.d. model. It is dispensable in the i.d. and i.n. models in the spleen. Thus, we can conclude that the identification of immune markers associated with protection against infection is strongly affected by the route of infection used and the organ analyzed.

## Discussion

There are well-documented cases in which generalization of use of a new drug favors, in just a few decades, the development of resistance leading to treatment failure [reviewed in Zur Wiesch et al. (55)]. This problem has become so acute that drug resistance is viewed as one of the major challenges in public health, reinforcing the importance of vaccines in the control of epidemics.

Historically, highly successful global vaccination campaigns have been associated with the administration of live-attenuated vaccines (LAVs) (1). Today, modern vaccination strategies focus mainly on the use of subunit vaccines (SUVs) with an adjuvant (56–58). Compared to LAVs, SUVs offer several advantages: (i) their supposed safety because they exclude all risks of reversion of attenuated pathogens to a virulent form, (ii) their high specificity limiting the risk of autoimmune diseases, and (iii) the ease to produce, conserve, and transport them. But the benefits of SUVs hide some weaknesses. In addition to their classical antigen- or target-specific protective effects, LAVs can induce non-specific protective effects. Epidemiological data suggest that vaccination with smallpox, measles, BCG or oral polio vaccines results in increased overall childhood survival [reviewed and discussed in Benn et al. (59) and de Bree et al. (60)]. These observations can be partially explained by the poly-specificity of antigenic T and B receptors, the Mackaness effect and the induction of innate immune memory (trained immunity) (61). SUVs drive immune responses against a reduced number of dominant antigens and are associated with a modern adjuvant selected for its low inflammatory potential. Because this design reduces the possibilities of TCR and BCR-mediated cross-protection and a short-lived, low level of activation of innate immune response, it limits the possibility of non-specific protection. More importantly, vaccine resistance, although rare, is now well-documented for several SUVs (62). This seems mainly due to single mutational events, suggesting that small variations of pathogens can allow them to escape to sharp antigen selection pressure generated by SUVs. These weaknesses of SUVs, combined with the growth of antibiotic resistance, could in the near future leave us extremely helpless in the face of epidemics.

One solution to this problem is the rational development of second-generation LAVs retaining the efficiency of first-generation LAVS but compatible with current safety standards. This goal is achievable thanks to our better understanding of the genetics of microorganisms. However, we lack a rigorous methodology for selecting candidate vaccines in animal models.

Ideally, an effective and safe LAV should induce strong and adapted mucosal and systemic immunity against the target pathogen, without persisting or disseminating in the host. This requires, in particular, that the natural niche of pathogen persistence, the virulence genes necessary for pathogen persistence and the immune markers associated with the protective memory state be identified. The vast majority of studies attempting to identify these factors in animal experimental models are carried out using a single route of infection. Often, for historical or convenience reasons, a single route of infection is considered as the reference. In the case of studies to select candidate vaccines against *Brucella* in mice models, the reference is the intraperitoneal (i.p.) route (63) that leads to a rapid systemic infection. Rare studies use the more physiological intranasal (i.n.) or oral infection routes. The purpose of the present study was to highlight the impact of the route of infection on the identification of infection niches, virulence genes and immune markers associated with the development of a protective immune memory in the *Brucella* model.

We took advantage of the fact that we have already studied the reservoir of infection and the type of protective immune response in the i.p. (19, 20, 30) and i.n. (21, 36) *Brucella* infection models. In order to include a third model of infection in our comparison, we developed and characterized an original model of intradermal (i.d.) infection in this study. Contamination by cutaneous route is not the most common for *Brucella*, but it is however well-documented in certain occupational groups (13–15). We have chosen to characterize this route, rather than a more common type of *Brucella* contamination such as the oral route, because of its technical simplicity and because a large number of vaccines are given subcutaneously, including some *Brucella* vaccines in animals.

We observed that, following i.d. infection, *Brucella* disseminates immediately into the spleen and liver through the bloodstream and reaches the popliteal draining LNs in 24 h. *Brucella* is detected later in the mediastinal lymph nodes, muscles, and brain. At 50 days post infection, *Brucella* persists only in the spleen and LNs. As expected, control of the primary cutaneous infection requires a functional IL-12_p35_/IFNγ axis, TNFα, as well as CD4^+^T cells. CD8^+^ T cells, γδ^+^T cells, B cells and IL-17RA deficiencies appear to be dispensable. Thus, the i.d. model seems to be similar to the i.p. model (19). It differs strongly from the i.n. infection model which requires γδ^+^T cells and IL-17RA to control *Brucella* during the early phase of lung infection (21).

The i.d. model is characterized by recruitment of monocytes and neutrophils at sites of skin infection in association with transitory moderate swelling. A deficiency of CCR2 results in a delay in the recruitment of monocytes and a transitory lack of *Brucella* control at the cutaneous lesion and popliteal draining LN. IFNγR deficiency also reduces monocyte recruitment and is associated with a strong influx of neutrophils in the footpad lesion, development of necrosis and a high CFU count in the lesion, LN, and spleen. In contrast, as previously reported by us (21), in the i.n. *Brucella* infection model, monocyte and neutrophil recruitment is not observed in the lung and CCR2 deficiency does not affect the control of infection. Interestingly, in the i.d. model, CCR7 deficiency leads to a significant reduction of the spread of *Brucella* from the footpad lesion to the draining LN and the spleen at 1 and 3 days post infection, and an important increase in the number of bacteria in the cutaneous lesion at 7 and 14 days. Taken together, our data suggest that recruited monocytes play a key role in the control of *Brucella* in the i.d. model. They also suggest that dissemination of *Brucella* from the primary lesion to the draining LN and spleen could implicate CCR7-dependent cell migration. In agreement, Archambaud et al. (37) have shown that *Brucella* can migrate from the lung to draining LNs by infecting dendritic cells and alveolar macrophages.

An original and surprising observation in the i.d. infection model is the inability of the protective memory response to prevent establishment of *Brucella* in popliteal draining lymph nodes. The memory response eliminates *Brucella* in the cutaneous lesion, blood, and spleen, but fails to control *Brucella* in the draining LN, suggesting that there is a special reservoir protecting *Brucella* in this tissue. These LN reservoir cells should be better characterized, as von Bargen et al. (64) reported that LNs constitute the first site of *Brucella* infection and multiplication during oral infection in mice and humans. Unfortunately, in our i.d. model, the low CFU counts in LNs at later times of infection did not allow us to analyse the LN reservoir cells by flow cytometry and made it very difficult to visualize them by fluorescence microscopy.

By comparing i.p., i.n., and i.d. models, we observed that the host-pathogen relationship is strongly affected by the route of infection.

First, the pattern of tissue infection appears to vary widely depending on the route of infection. The i.p. route of infection leads to immediate systemic dissemination of *Brucella* to all tissues tested. In contrast, i.n. infection displays a more restricted pattern of infected tissues, including mainly the lung, mediastinal draining LNs and spleen. The i.d. model presents an intermediate pattern, with systemic dissemination to the spleen and liver and localized strong persistence of *Brucella* in the cutaneous lesion and popliteal draining LNs. The impact of the route of infection on the spread of bacteria has already been documented by whole body imaging in mice with radiolabelled or bioluminescent bacteria. However, only high bacteria levels in tissues is detected by this approach. Consequently, high doses of bacteria need to be administered and weakly infected organs are not identified. In the *Francisella* model (65), 2 × 10^9^ radiolabelled bacteria were administrated and their dissemination was only monitored until 20 h post infection. In models using bioluminescent *Brucella melitensis* (66) and *Brucella suis* (67), 1 × to 2.5 × 10^7^ bacteria were injected. In the spleen of mice infected with 2.5 × 10^7^ CFU of *B. melitensis*, no bioluminescence was detected despite the detection of 4.8 × 10^3^ CFU by plating (66), confirming the very poor sensitivity of bioluminescence detection. In contrast, though time-consuming, our classical approach based on the plating of select tissues detects up to 10 bacteria per tissue and allows us to use a moderate dose of infection, 2 × 10^4^ CFU per mouse. Our results showed that, at 50 days post infection, *Brucella* persists mainly in lymphoid organs, such as the spleen and LNs in the three models, but also in the thymus in the i.p. model. However, our goal here was not to identify all possible *Brucella* persistence sites in mice and *Brucella* may persist in other tissues, such as the bone marrow as recently described (68).

Second, using eFluor670 labeled *Brucella*, we demonstrated that the type of cells infected first depends on the route of infection. As previously observed, we confirmed that the main cells infected in the lung of i.p. and i.n. infected mice at 2 and 24 h post infection were F4/80^+^ red pulp macrophages (30) and alveolar macrophages (37), respectively. Surprisingly, we observed that, in addition to F4/80^+^ monocytes/macrophages, Ly6G^+^ neutrophils and CD45^neg^ CD140a^+^ fibroblasts also appear to be infected in the footpad lesion of i.d. infected wild-type mice. *Brucella*-infected neutrophils have been described in pathological conditions (69). To our knowledge, this is the first description of *Brucella*-infected fibroblasts *in vivo* in wild-type mice. Infection by *Brucella* of neutrophil and fibroblasts in the footpad lesions was confirmed by confocal microscopy. The infection of different cell types can have a significant impact on the persistence of *Brucella*. It has been observed *in vitro* that the intracellular trafficking of *Brucella* is dependent on the type of cells infected. For example, *Brucella abortus* replicates in a vacuole derived from the LAMP1^neg^ endoplasmic reticulum in epithelial cells, macrophages and dendritic cells, although in extravillous trophoblasts it replicated within single-membrane acidic LAMP1^pos^ inclusions (70).

Third, we observed that the route of infection affects the identification of a major virulence gene. The Δ*virB Brucella* strain, known to be attenuated in the i.p. infection model but able to persist for several weeks in the spleen (39), is unable to persist beyond 48 h in the lung in the i.n. infection model but, surprisingly, persists longer than the wild-type strain in cutaneous lesions in the i.d. infection model. This unexpected result is probably due in part to the different type of cells infected. It has been reported that ΔwadC (unable to encode LPS core glysosyltransferases) *Brucella abortus* multiplies in bone marrow-derived macrophages, RAW264.7 macrophages or HeLa cells but is killed in bone marrow-derived dendritic cells (71). Microscopic analysis showed that the Δ*virB Brucella melitensis* strain multiplies very weakly in alveolar macrophages, ~15-fold less than the wild type strain. In striking contrast, it multiplies at the same level as the wild-type strain in splenic macrophages and at an intermediate level in dermal cells, ~2-fold less than the wild type strain. This slightly lower multiplication rate of the Δ*virB Brucella* strain in the footpad lesion may make *Brucella* more stealthy and thus less detectable by the immune system and able to persist longer than the wild type strain. Taken together, our data lead us to conclude that a bacterial strain can be considered as attenuated or not depending on the route of infection and the tissues analyzed.

Fourth, we demonstrate that the route of infection also affects the identification of immune markers associated with a protective memory response. As summarized in Figure 15, which compares the results obtained in this article in the i.d. infection model as well as those obtained previously by us in the i.p. (20) and i.n. (21) infection models, *Brucella* control in the spleen in challenge conditions required different lymphocyte subsets and T helper responses depending on the route of infection. Control in the i.p. model appears to be dependent on IL-12_p35_, CD4^+^T cells and B cells (20). In the i.n. model, only αβ^+^T cells appear to be strictly required. A deficiency of CD4^+^T cells, CD8^+^T cells, B cells or IL-12_p35_ has no significant impact on *Brucella* control in the spleen. Only the simultaneous deficiency of IL-12_p35_ and IL17RA leads to a lack of control (21). Finally, in the i.d. infection model, B cells appear to be indispensable, like in the i.p. model, but CD4^+^T cells are required in the lesion but not in the spleen. More surprisingly, *Brucella* control in the lesion required IL-17RA, whereas it was not necessary for control of the primary infection. And conversely, IL-12_p35_, which is needed to control the primary infection, is no longer essential to control the challenge in the lesion.

It may seem surprising that, depending on the route of infection, control of *Brucella* in the same organ, the spleen, requires different types of lymphocyte populations and different T helper responses. These different needs may be the consequence of the specific immune conditions prevailing at the primary infection site in the different models. It is well-established that the composition of immune and stromal cells, as well as the phenotype of those cells, the nature of the microenvironment and the isotypes of the antibodies differ in each tissue and affect the ability of the immune system to control both infections and tumor growth [reviewed in Engwerda et al. (72) and Pao et al. (73)]. Therefore, depending on the route of infection and on the primary infection site, the bacterial load reach in the blood and spleen may differ significantly. We observed this in practice when comparing our 3 models (Figure 1D). The need for B cells is correlated with the rapid spread of bacteria in the blood, like in the i.p. and i.n. models. In the i.n. model, B cells are not needed and *Brucella* is not detected in the blood at any time. The same reasoning can be applied to the requirement for IL-12p35. IL-12p35 is indispensable in the spleen only in the i.p. model, where the bacterial load reaching the spleen is the highest. Regardless of the dose reaching the organ over time, *Brucella* could also reach the spleen in different forms depending on the route of infection. During the first 24 h following i.p. infection, *Brucella* is found in the blood in extracellular form (35). In the i.n. model, *Brucella* disseminates from the lung to draining lymph nodes inside dendritic cells and alveolar macrophages (37). Therefore, *Brucella* could reach the spleen by being protected from antibodies within a cell, which would explain that B cells are not necessary.

The route of infection has been reported to impact the nature of the protective immune response in certain other infection models. For example, control of an i.p. *Listeria monocytogenes* challenge strictly requires memory CD4^+^ and CD8^+^ αβ^+^T cells producing IFNγ (74). But in the oral *L. monocytogenes* infection model, intestinal multifunctional γδ^+^T cells able to simultaneously produce IFNγ and IL-17A can provide enhanced protection against infection and even compensate for the absence of αβ^+^T cells (75). However, the impact of different routes of infection on the type of protective memory response is rarely compared and should be more systematic in view of its importance.

One of the major interests of our comparison of the i.p., i.n., and i.d. infection routes is that all of our experiments were conducted using the same strains of *Brucella melitensis* 16M and C57BL/6 mice, with the same infectious dose and under the same animal facility conditions. There is a large body of available data on mice infected i.p. or i.n. by *Brucella* in the literature. It is very difficult, however, to truly compare the data from these models because the infectious doses and the strains of *Brucella* and mice are often different. For example, the study of Hielpos et al. (76) showed that *B. abortus* persisted at high levels in the lungs for at least 7 days and was detected in the spleen as early as 24 h p.i., much earlier than in our i.n. model of infection. However, BALB/c mice, that are more susceptible to *Brucella* than the C57BL/6 strain, were used in the Hielpos study and were infected with 10^6^ CFU of *B. abortus*, at a dose 50 times higher than our infection dose. Under these experimental conditions, it is therefore quite normal that *Brucella* was detected in the spleen much faster than in our model. Even though the experimental conditions seem similar, it is well-known that, depending on the animal facility conditions, the immunological experience of mice can affect their ability to control an infection non-specifically and that a strain of bacteria can drift in the laboratory and present slightly different virulence. Consequently, comparisons between different models of *in vivo* infections that are not performed under exactly the same experimental conditions are often hazardous.

It is important to point out that, since the mouse is not the natural host of *Brucella melitensis*, we do not consider that one particular route of infection in a mice model is more physiological or informative than another regarding what is happening in the natural host during a *Brucella* infection. This work must be seen as fundamental research work carried out with the aim of improving the methodology for the rational design of LAVs in animal models. However, our comparison of three routes of infection allows us to draw some practical conclusions. We observed that both the humoral response and the cellular response are essential to the control of a challenge in the i.p. and i.d. models, which is not the case in the i.n. model where a humoral response and even αβT cells are dispensable. As discussed previously, this can be explained by the fact that the i.p. and i.d. routes of infection lead to massive dissemination of *Brucella* in the blood, which is not the case for the i.n. route. Likewise, in sheep, inoculation by the subcutaneous route produced wider and more generalized infections than the conjunctival route (50). Thus, the i.p. and i.d. routes lead to systemic infection that therefore appears to be more difficult to control than a mucosal i.n. infection and consequently may be a more demanding test in mice for assessing the protective capacity of a vaccine candidate. If we compare i.p. and i.d. models, the latter display the advantage of being close to the subcutaneous vaccination route conventionally used with the REV1 *B. melitensis* vaccine in small ruminants. The i.d. model of infection in mice also displays a pattern of tissue infection that is quite similar to that described in the goat (77) following subcutaneous infection with a virulent strain of *Brucella melitensis*. Thus, for these reasons, i.d. could constitute a new promising route of delivery for tests of candidate vaccines in a mouse model.

In summary, our study demonstrates that the identification of candidate LAVs and immune protection markers in an animal model can be strongly affected by the route of infection used. We therefore recommend that researchers systematically compare different routes of infection, identified as those closest possible to the natural host infection, and not be limited to the analysis of a single tissue type. As the infectious doses (78) as well as the strain of pathogen analyzed (79) can also strongly affect the type of protective immune response, as has been well-documented in the experimental mouse model of *Leishmania major* infection, we can conclude that the selection process of candidate LAVs is much more complex than expected.

## Data Availability

The raw data supporting the conclusions of this manuscript will be made available by the authors, without undue reservation, to any qualified researcher.

## Ethics Statement

The procedures used in this study and the handling of the mice complied with current European legislation (Directive 86/609/EEC) and the corresponding Belgian law Arrêté royal relatif à la protection des animaux d'expérience of 6 April 2010 and published on 14 May 2010. The Animal Welfare Committee of the Université de Namur (UNamur, Belgium) reviewed and approved the complete protocol for Brucella infection (Permit Number: UN-LE-13/195).

## Author Contributions

AD and EM wrote the article. AD, AL, AM, MV, and J-MV realized experiments. GP, XD, J-JL, and EM provided reagents.

### Conflict of Interest Statement

The authors declare that the research was conducted in the absence of any commercial or financial relationships that could be construed as a potential conflict of interest.

## Supplementary Material

Figure S1 Each route of infection leads to a specific pattern of infected organs (part 1). Wild-type C57BL/6 mice were infected intraperitoneally (i.p.), intranasally (i.n.) or intradermally (i.d.) with a dose of 2 × 10^4^ CFU of mCherry-*B. melitensis* and sacrificed at the indicated times. The data represent the CFU count per footpad lesion, popliteal LN, lung, or mediastinal LN. Gray bars represent the median. The significant differences between the indicated groups are marked with asterisks: ^*^*p* < 0.1, ^**^*p* < 0.01, ^***^*p* < 0.001. These results are representative of two independent experiments. LN, lymph node; h, hours; d, days; n, number of mice per group.

Figure S2 Each route of infection leads to a specific pattern of infected organs (part 2). Wild-type C57BL/6 mice were infected intraperitoneally (i.p.), intranasally (i.n.) or intradermally (i.d.) with a dose of 2 × 10^4^ CFU of mCherry-*B. melitensis* and sacrificed at the indicated times. The data represent the CFU count per skeletal muscle, heart, tail, brain, liver, or ovary. Gray bars represent the median. The significant differences between the indicated groups are marked with asterisks: ^*^*p* < 0.1, ^**^*p* < 0.01, ^***^*p* < 0.001. These results are representative of two independent experiments. h, hours; d, days; n, number of mice per group.

Figure S3 Intraperitoneal *Brucella* infection leads to infection of a large panel of lymph nodes. Wild-type C57BL/6 mice were infected intraperitoneally (i.p.) with a dose of 2 × 10^4^ CFU of mCherry-*B. melitensis* and sacrificed at 50 days post infection. The data represent the CFU count per lymphoid organs (spleen, right inguinal LN, right axillary LN, mediastinal LN, mesenteric LN, and right popliteal LN). Gray bars represent the median. These results are representative of two independent experiments. LN, lymph node; n, number of mice per group.

Figure S4 Impact of eFluor670 staining on *Brucella* growth *in vitro* and *in vivo*. **(A)** 4 × 10^4^ CFU/ml of mCherry-*B. melitensis* were prepared from an overnight liquid culture and stained with the eFluor670 fluorochrome. Bacteria were fixed and then analyzed by flow cytometry. **(B)** Comparison by Bioscreen analysis of the growth of mCherry-*B. melitensis* and eFluor670 stained mCherry-*B. melitensis* in rich medium (2YT). These data represent the mean of three independent experiments. **(C)** Wild-type C57BL/6 mice were infected i.n. with a dose of 2 × 10^4^ CFU of mCherry-*B. melitensis* or eFluor670 labeled mCherry-*B. melitensis* and sacrificed at 5 or 12 days post infection. The data represent the CFU count per lung. **(D)** Wild-type C57BL/6 mice were infected i.p. with a dose of 10^5^ CFU of mCherry-*B. melitensis* eFluor670 labeled mCherry-*B. melitensis* and sacrificed at 5 or 12 days post infection. The data represent the CFU count per spleen. These results **(C,D)** are representative of two independent experiments. Gray bars represent the median. d, days; n, number. The significant differences between the indicated groups are marked with asterisks: ^*^*p* < 0.1.

Figure S5 Phenotype of infected footpad lesion cells from wild type mice. Wild-type C57BL/6 mice were infected intradermally with 10^7^ CFU of *B. melitensis*. Control wild-type mice were injected with PBS. The footpad lesions were harvested at 2 or 24 h post infection and the cells were analyzed by flow cytometry. The data result from the flow cytometry analysis of eFluor670, CD45, CD140a, and CD11b expression on footpad cells. The data show the representative dot plot from individual mice. These results are representative of three independent experiments.

Figure S6 Phenotype of infected CD11b-positive footpad lesion cells from wild type mice. Wild-type C57BL/6 mice were infected intradermally with 10^7^ CFU of *B. melitensis*. Control wild-type mice were injected with PBS. The footpad lesions were harvested at 2 or 24 h post infection and the cells were analyzed by flow cytometry. The data result from the flow cytometry analysis of eFluor670, F4/80, Ly6G, and CD11b expression on footpad cells. The data show the representative dot plot from individual mice. These results are representative of three independent experiments.

Figure S7 Complemented Δ*virB* strain growth in the lung. Wild-type C57BL/6 mice were infected i.n. with a dose of 10^5^ CFU of wild type mCherry-*B. melitensis*, Δ*virB* mCherry- *B. melitensis* or complemented Δ*virB* mCherry-*B. melitensis* (Δ*virB* mCherry-*B. melitensis* p*virB*). Mice were sacrificed at 5 days post infection and the lung was collected. The data represent the CFU count per lung. Gray bars represent the median. Significant differences between the indicated groups are marked with asterisks: ^**^*p* < 0.01, ^***^*p* < 0.001. These results are representative of two independent experiments. n, number of mice per group.

Figure S8 eFluor670 labeling identified newborn *Brucella*. mCherry *Brucella melitensis* is labeled with eFluor670. **(A)** Schematic representation of unipolar growth of eFluor670 labeled mCherry-*Brucella*. As eFluor670 does not move in the bacterial membrane, the newly formed bacterium, called the newborn, loses the eFluor670 labeling, allowing its identification by fluorescent microscopy. **(B)** Representative image at 0 and 24 h of eFluor670 labeled mCherry-*Brucella* extracellular growth *in vitro*. The 24 h image shows a division and a newborn cell (mCherry^+^ eFluor670^−^). The panels are color-coded with the text for mCherry and eFluor670. Scale bar = 5 μm. h, hours; n, newborn; pos, positive; neg, negative.

Figure S9 BALB/c mice are more susceptible to cutaneous *Brucella* infection than C57BL/6 mice. Wild-type C57BL/6 and BALB/c mice were infected intradermally with a dose of 2 × 10^4^ CFU of *B. melitensis* and sacrificed at the indicated times. The data represent the CFU count per organ. Gray bars represent the median. The significant differences between the indicated groups are marked with asterisks: ^*^*p* < 0.1, ^**^*p* < 0.01, ^***^*p* < 0.001. These results are representative of two independent experiments. LN, lymph node; d, days; n, number of mice per group.

Figure S10 Neutrophils constitute the major population in the footpad lesion from infected IFNγR^−/−^ mice. Wild-type, IFNγR^−/−^, and CCR2^−/−^ C57BL/6 mice were infected intradermally with a dose of 2 × 10^4^ CFU of mCherry-*B. melitensis*. Control wild-type mice were injected with PBS. The footpad lesions were harvested at 3 and 7 days post infection and the cells were analyzed by flow cytometry. **(A)** Flow cytometry analysis of CD11b, F4/80, and LY6G expression on footpad cells. The data show the representative dot plot from individual mice. **(B)** Data represent the mean frequency (*n* = 4) of neutrophils and monocytes in the footpad lesion at the indicated time of infection. These results are representative of three independent experiments. Cont, control; d, days.

Figure S11 Humoral immune response induced by intradermal *Brucella* infection. Wild-type C57BL/6 mice were infected intradermally **(A)** or intraperitoneally **(B)** with a dose of 2 × 10^4^ CFU of mCherry-*B. melitensis*. Serum was collected at the indicated times, and ELISA was performed to determine the isotype distribution of the *Brucella*-specific antibodies. The data represent the means ± SEM of results for 8 mice. These results are representative of three independent experiments. O.D, optical density; d, days.

Figure S12 Comparison of protection in wild-type and various deficient mice previously immunized by intraperitoneal route with live *B. melitensis*. Wild-type, TCRγδ^−/−^, MHCII^−/−^, TAP1^−/−^, Il-12p35^−/−^, and IL-17RA^−/−^ C57BL/6 mice were immunized i.p. with 2 × 10^4^ CFU of live wild-type *B. melitensis* and treated with antibiotics, as described in the Materials and Methods. Naive (primary infection group) and immunized (secondary infection group) mice were challenged i.p. with 2 × 10^4^ CFU of live mCherry-*B. melitensis* and sacrificed at 12 days post infection. The data represent the CFU count per spleen. Gray bars represent the median. The significant differences between the indicated groups are marked with asterisks: ^**^*p* < 0.01. These results are representative of two independent experiments. n, number of mice per groups.
