# Supplementary figures and images for: Route of Infection Strongly Impacts the Host-Pathogen Relationship
Source: Front Immunol. 2019 Jul 11;10:1589. doi: 10.3389/fimmu.2019.01589 (PMC6637429; doi:10.3389/fimmu.2019.01589)

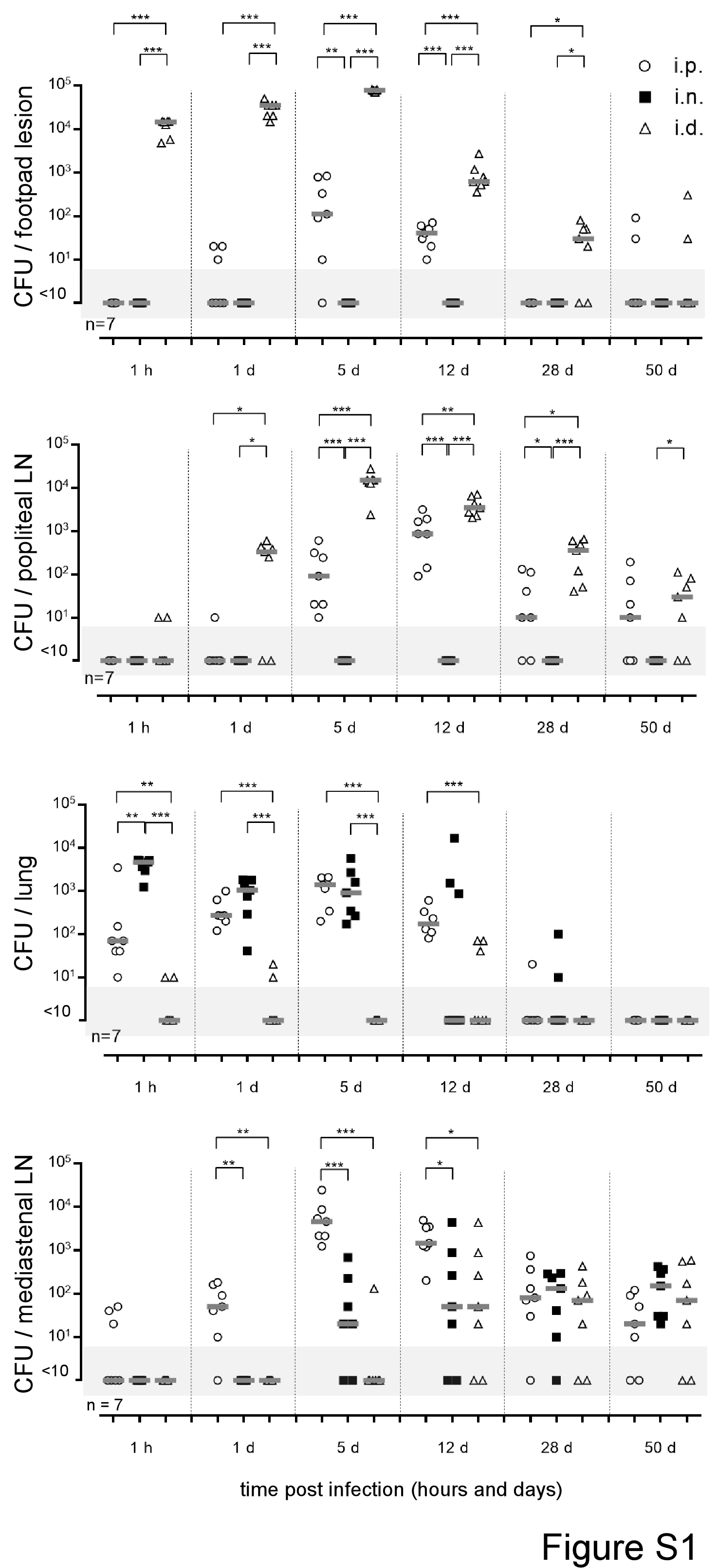

Supplement: Figure S1 — Each route of infection leads to a specific pattern of infected organs (part 1). Wild-type C57BL/6 mice were infected intraperitoneally (i.p.), intranasally (i.n.) or intradermally (i.d.) with a dose of 2 × 10^4^ CFU of mCherry-B. melitensis and sacrificed at the indicated times. The data represent the CFU count per footpad lesion, popliteal LN, lung, or mediastinal LN. Gray bars represent the median. The significant differences between the indicated groups are marked with asterisks: *p < 0.1, **p < 0.01, ***p < 0.001. These results are representative of two independent experiments. LN, lymph node; h, hours; d, days; n, number of mice per group. [file Image_1.TIF]

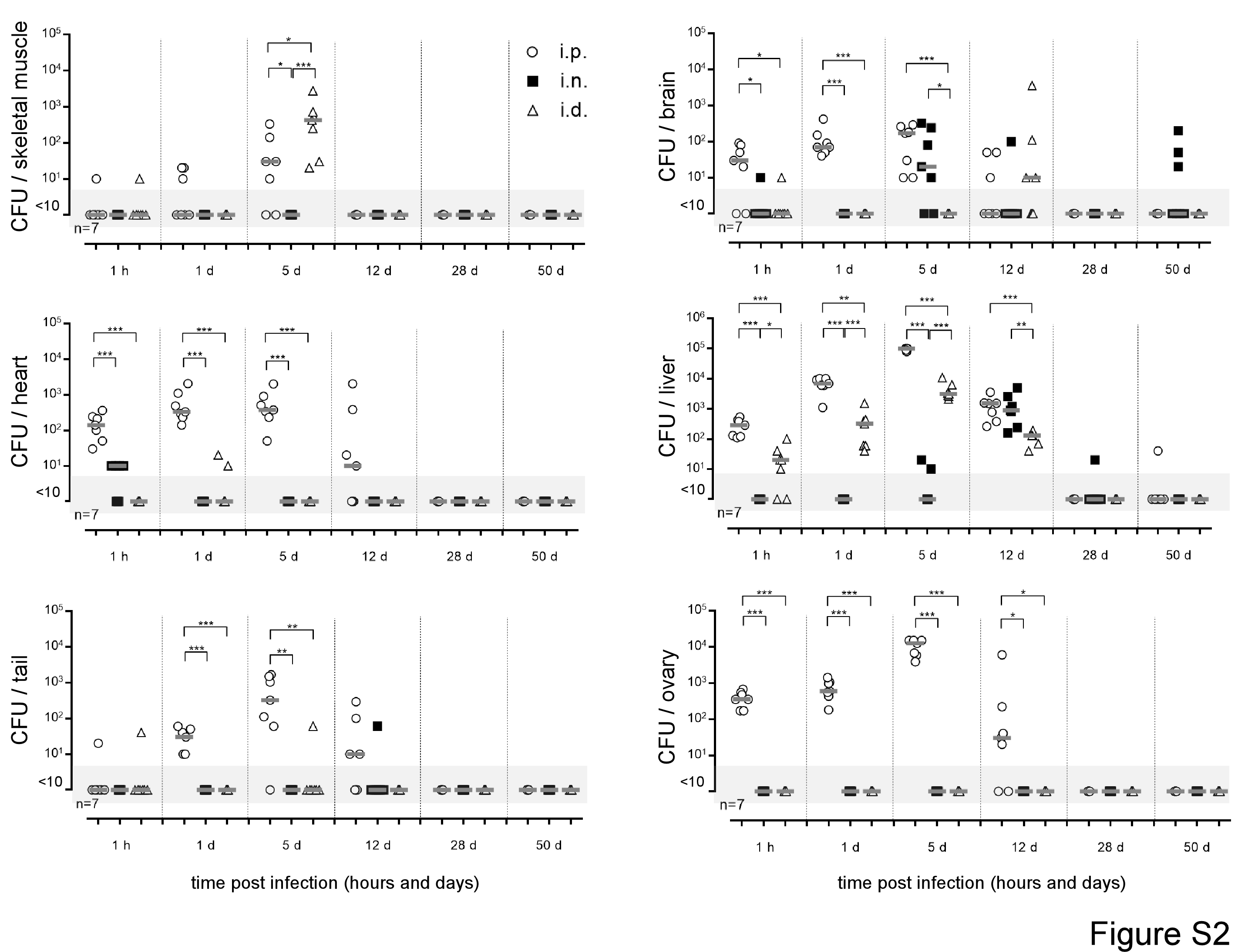

Supplement: Figure S2 — Each route of infection leads to a specific pattern of infected organs (part 2). Wild-type C57BL/6 mice were infected intraperitoneally (i.p.), intranasally (i.n.) or intradermally (i.d.) with a dose of 2 × 10^4^ CFU of mCherry-B. melitensis and sacrificed at the indicated times. The data represent the CFU count per skeletal muscle, heart, tail, brain, liver, or ovary. Gray bars represent the median. The significant differences between the indicated groups are marked with asterisks: *p < 0.1, **p < 0.01, ***p < 0.001. These results are representative of two independent experiments. h, hours; d, days; n, number of mice per group. [file Image_2.TIF]

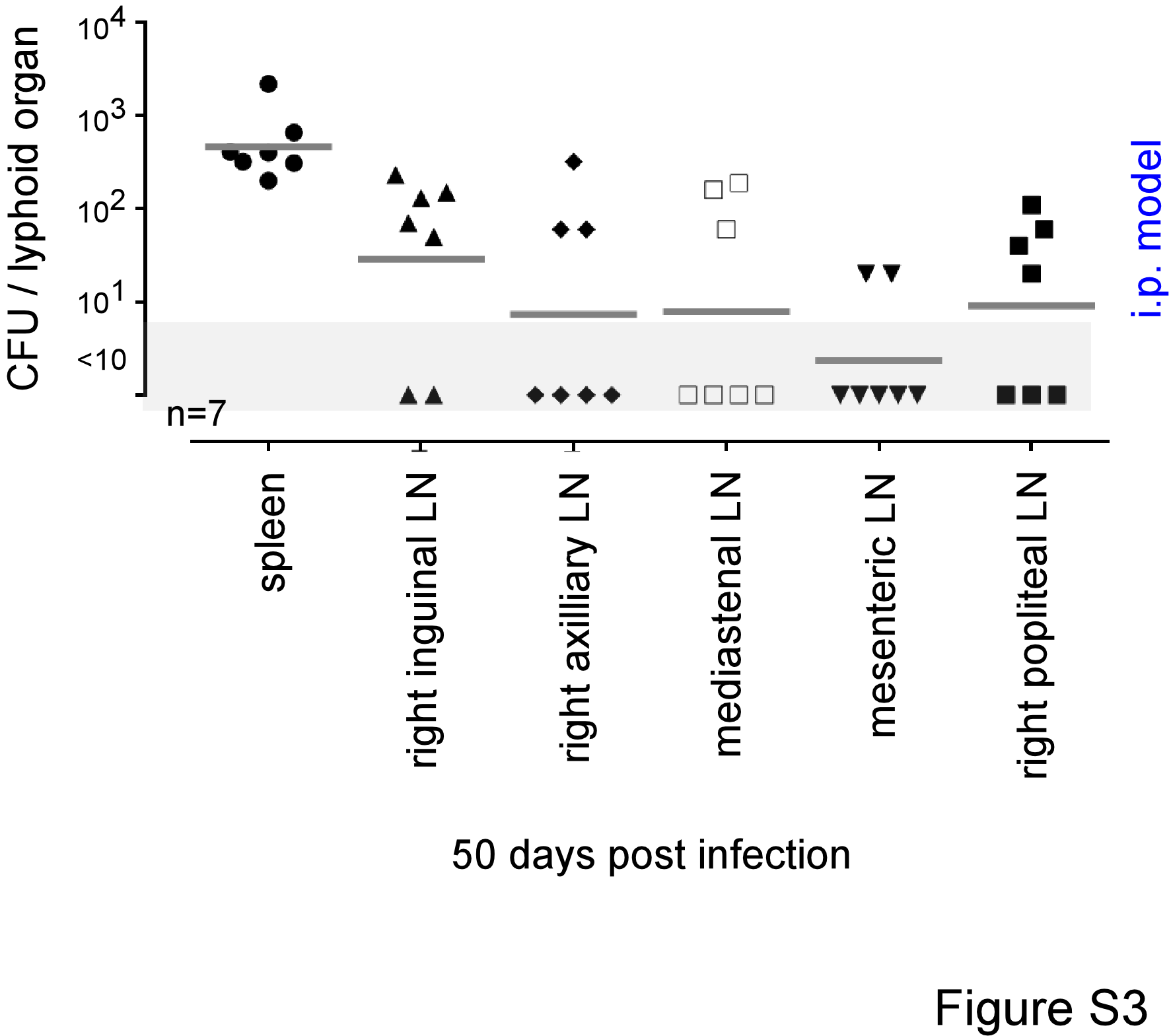

Supplement: Figure S3 — Intraperitoneal Brucella infection leads to infection of a large panel of lymph nodes. Wild-type C57BL/6 mice were infected intraperitoneally (i.p.) with a dose of 2 × 10^4^ CFU of mCherry-B. melitensis and sacrificed at 50 days post infection. The data represent the CFU count per lymphoid organs (spleen, right inguinal LN, right axillary LN, mediastinal LN, mesenteric LN, and right popliteal LN). Gray bars represent the median. These results are representative of two independent experiments. LN, lymph node; n, number of mice per group. [file Image_3.TIF]

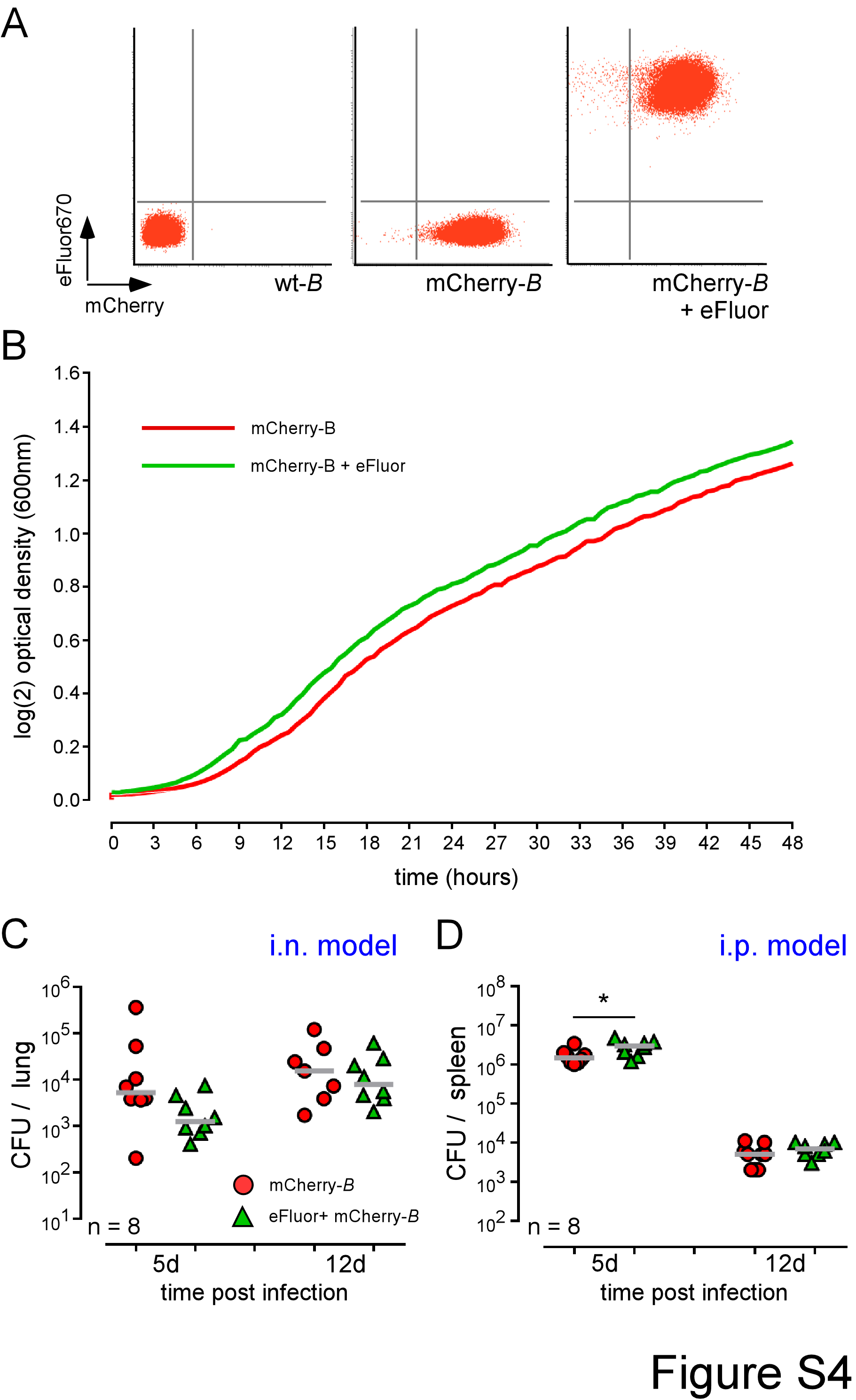

Supplement: Figure S4 — Impact of eFluor670 staining on Brucella growth in vitro and in vivo. (A) 4 × 10^4^ CFU/ml of mCherry-B. melitensis were prepared from an overnight liquid culture and stained with the eFluor670 fluorochrome. Bacteria were fixed and then analyzed by flow cytometry. (B) Comparison by Bioscreen analysis of the growth of mCherry-B. melitensis and eFluor670 stained mCherry-B. melitensis in rich medium (2YT). These data represent the mean of three independent experiments. (C) Wild-type C57BL/6 mice were infected i.n. with a dose of 2 × 10^4^ CFU of mCherry-B. melitensis or eFluor670 labeled mCherry-B. melitensis and sacrificed at 5 or 12 days post infection. The data represent the CFU count per lung. (D) Wild-type C57BL/6 mice were infected i.p. with a dose of 105 CFU of mCherry-B. melitensis eFluor670 labeled mCherry-B. melitensis and sacrificed at 5 or 12 days post infection. The data represent the CFU count per spleen. These results (C,D) are representative of two independent experiments. Gray bars represent the median. d, days; n, number. The significant differences between the indicated groups are marked with asterisks: *p < 0.1. [file Image_4.TIF]

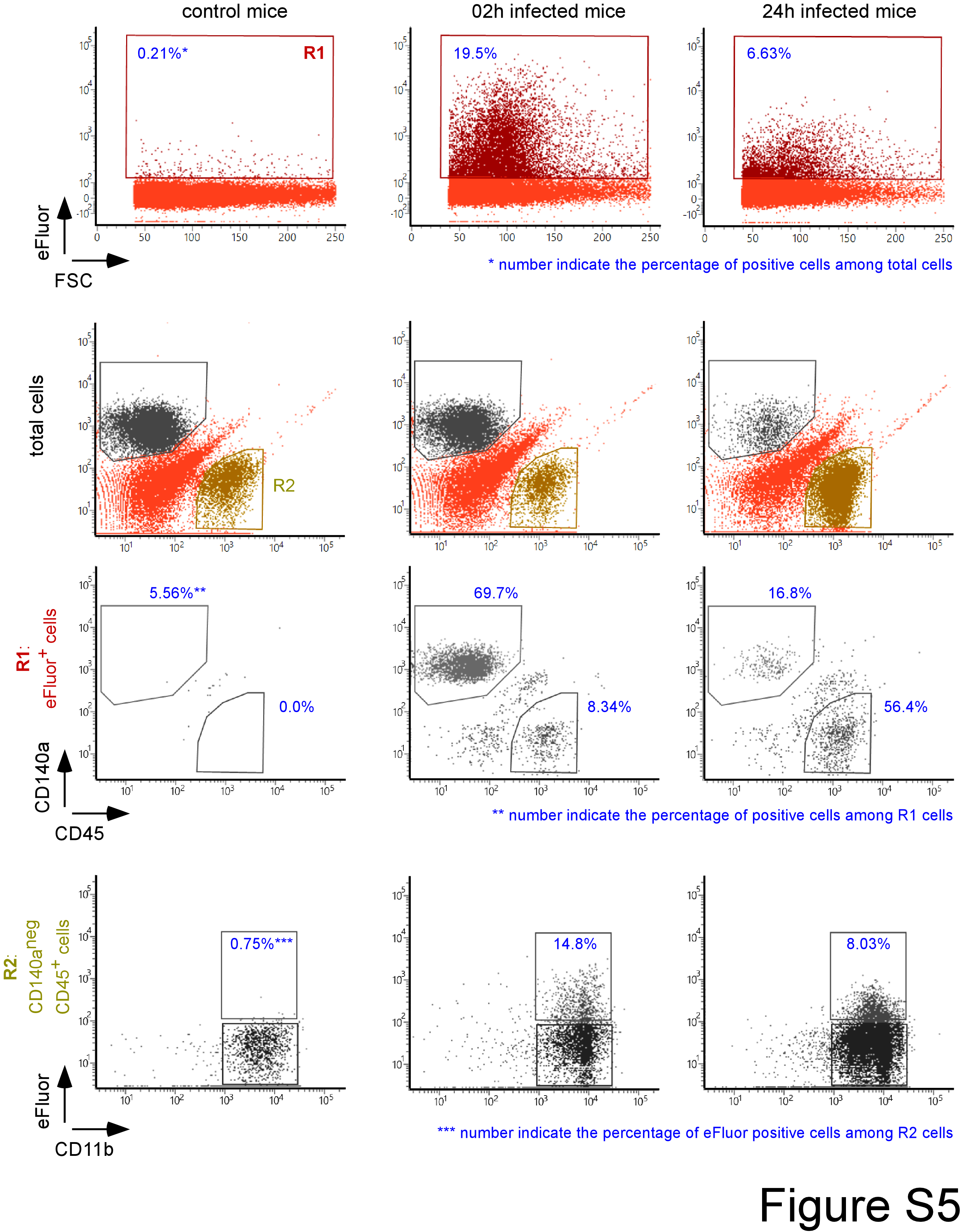

Supplement: Figure S5 — Phenotype of infected footpad lesion cells from wild type mice. Wild-type C57BL/6 mice were infected intradermally with 107 CFU of B. melitensis. Control wild-type mice were injected with PBS. The footpad lesions were harvested at 2 or 24 h post infection and the cells were analyzed by flow cytometry. The data result from the flow cytometry analysis of eFluor670, CD45, CD140a, and CD11b expression on footpad cells. The data show the representative dot plot from individual mice. These results are representative of three independent experiments. [file Image_5.TIF]

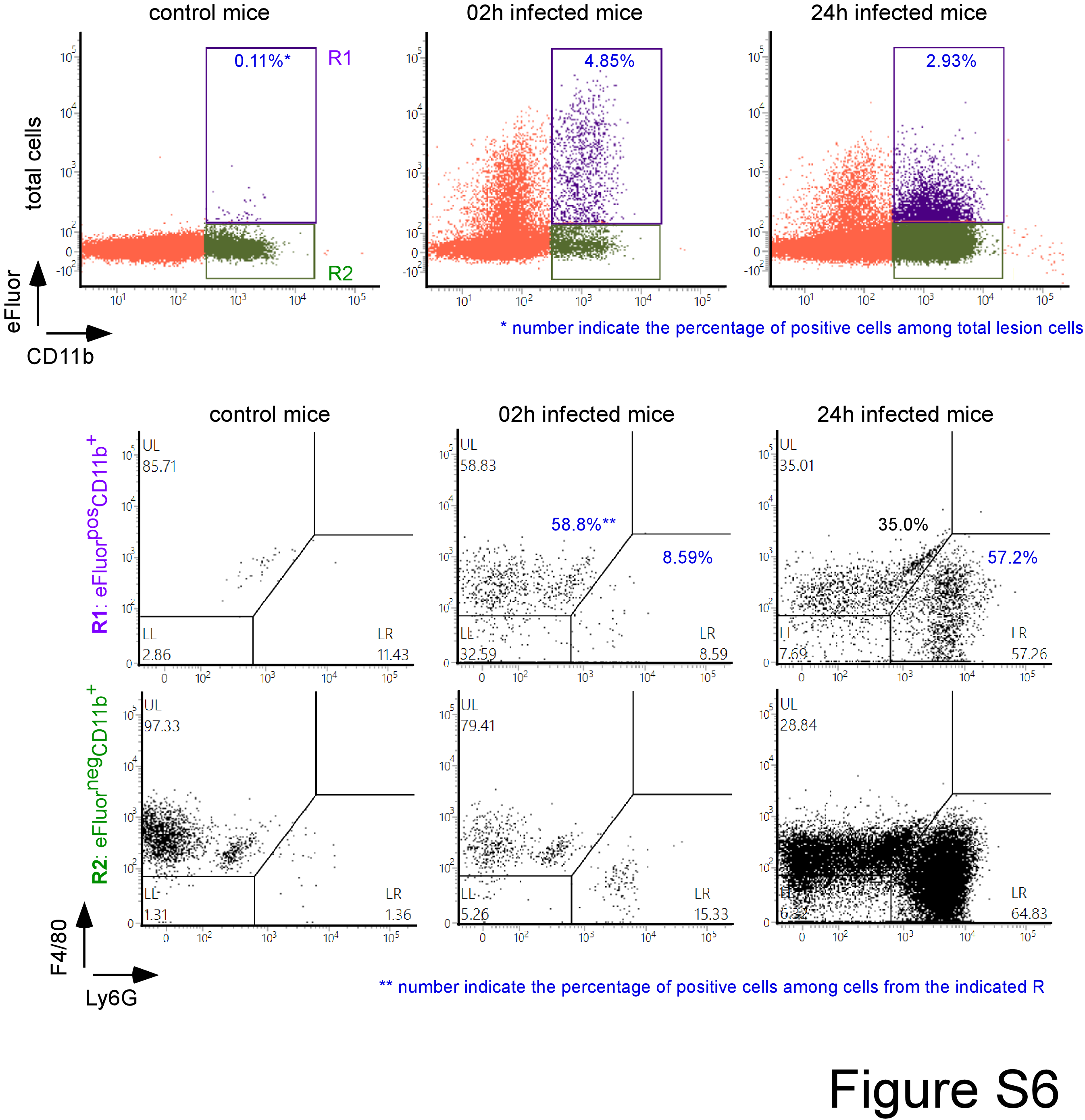

Supplement: Figure S6 — Phenotype of infected CD11b-positive footpad lesion cells from wild type mice. Wild-type C57BL/6 mice were infected intradermally with 107 CFU of B. melitensis. Control wild-type mice were injected with PBS. The footpad lesions were harvested at 2 or 24 h post infection and the cells were analyzed by flow cytometry. The data result from the flow cytometry analysis of eFluor670, F4/80, Ly6G, and CD11b expression on footpad cells. The data show the representative dot plot from individual mice. These results are representative of three independent experiments. [file Image_6.TIF]

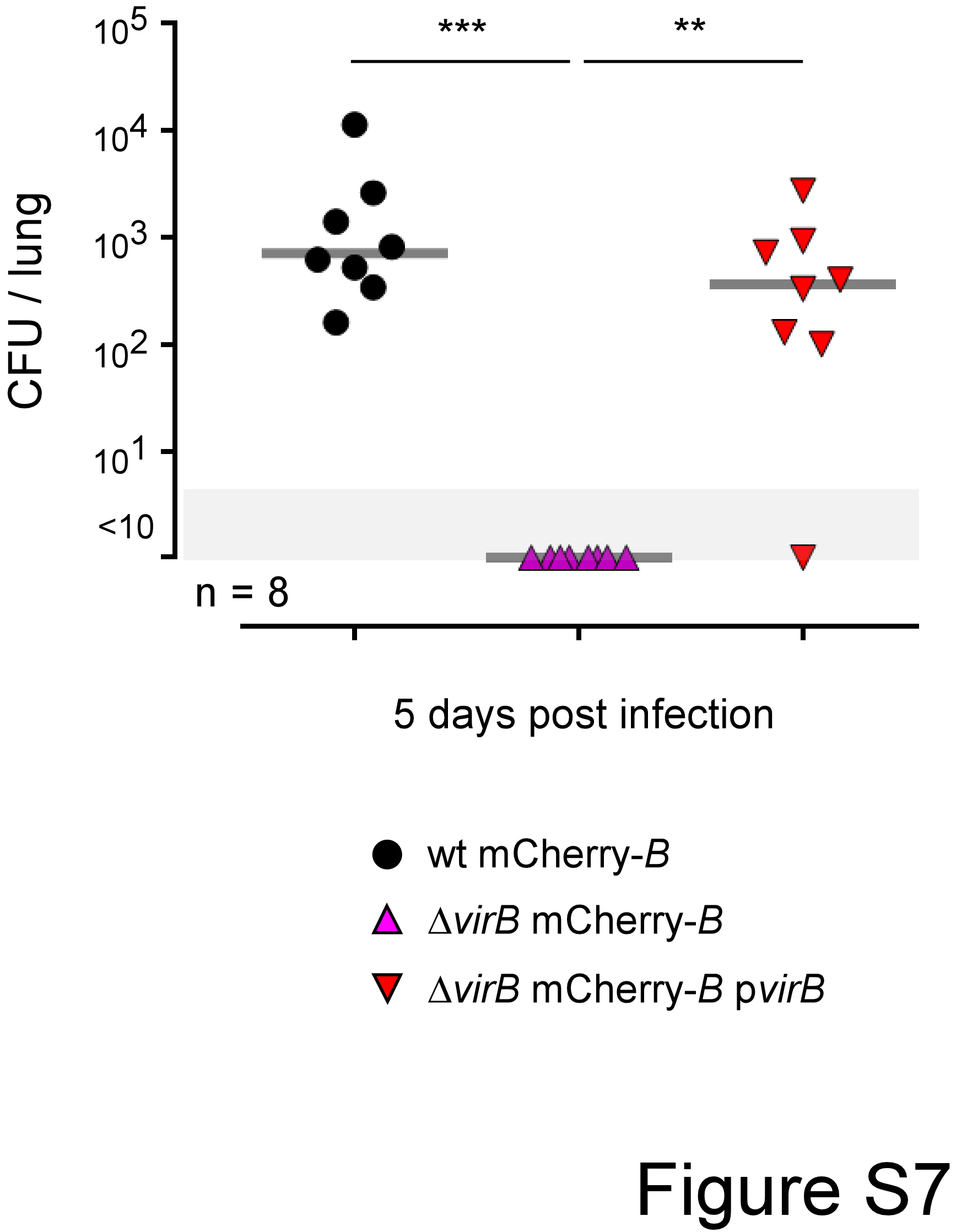

Supplement: Figure S7 — Complemented ΔvirB strain growth in the lung. Wild-type C57BL/6 mice were infected i.n. with a dose of 105 CFU of wild type mCherry-B. melitensis, ΔvirB mCherry- B. melitensis or complemented ΔvirB mCherry-B. melitensis (ΔvirB mCherry-B. melitensis pvirB). Mice were sacrificed at 5 days post infection and the lung was collected. The data represent the CFU count per lung. Gray bars represent the median. Significant differences between the indicated groups are marked with asterisks: **p < 0.01, ***p < 0.001. These results are representative of two independent experiments. n, number of mice per group. [file Image_7.TIF]

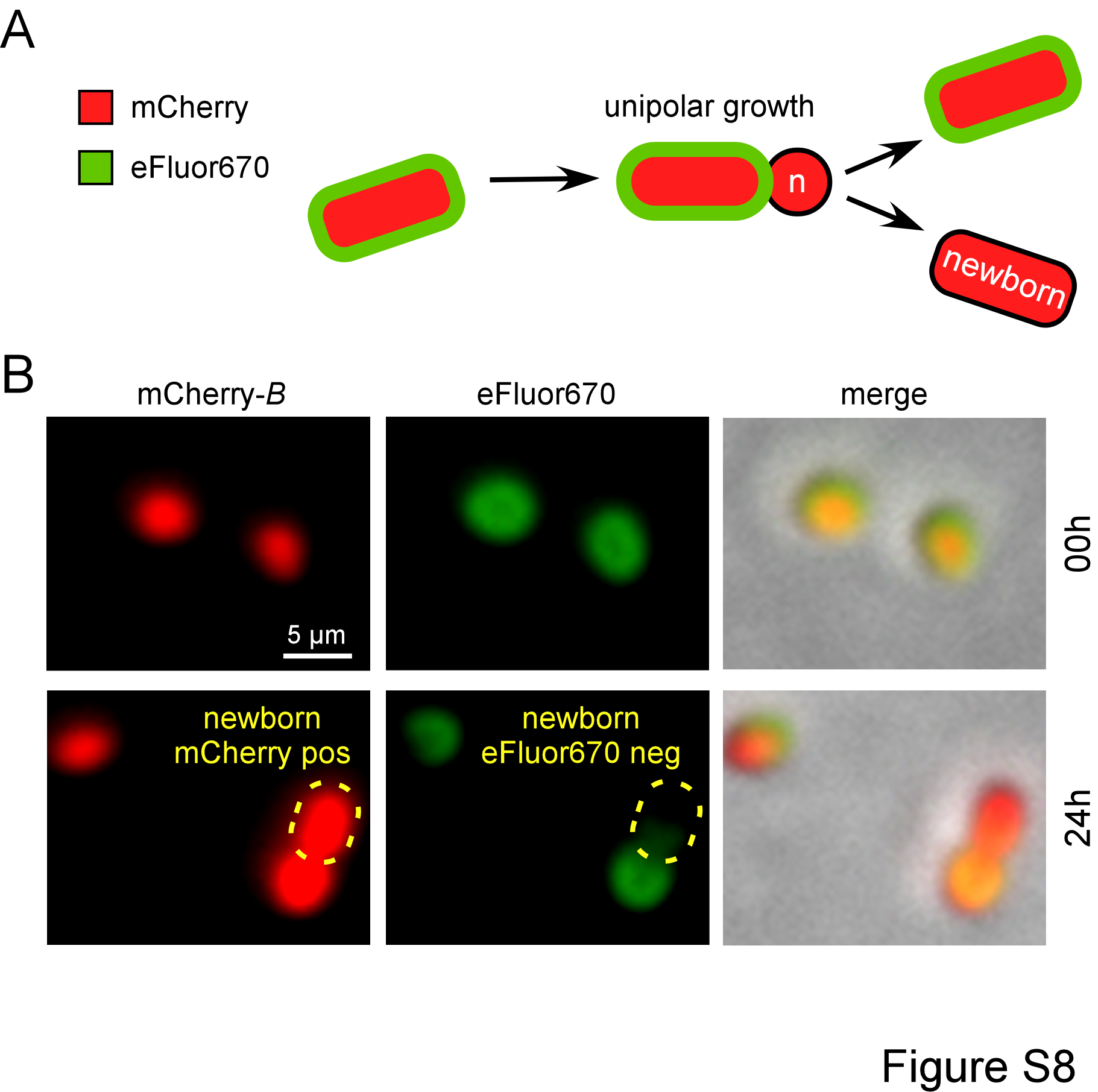

Supplement: Figure S8 — eFluor670 labeling identified newborn Brucella. mCherry Brucella melitensis is labeled with eFluor670. (A) Schematic representation of unipolar growth of eFluor670 labeled mCherry-Brucella. As eFluor670 does not move in the bacterial membrane, the newly formed bacterium, called the newborn, loses the eFluor670 labeling, allowing its identification by fluorescent microscopy. (B) Representative image at 0 and 24 h of eFluor670 labeled mCherry-Brucella extracellular growth in vitro. The 24 h image shows a division and a newborn cell (mCherry+ eFluor670−). The panels are color-coded with the text for mCherry and eFluor670. Scale bar = 5 μm. h, hours; n, newborn; pos, positive; neg, negative. [file Image_8.TIF]

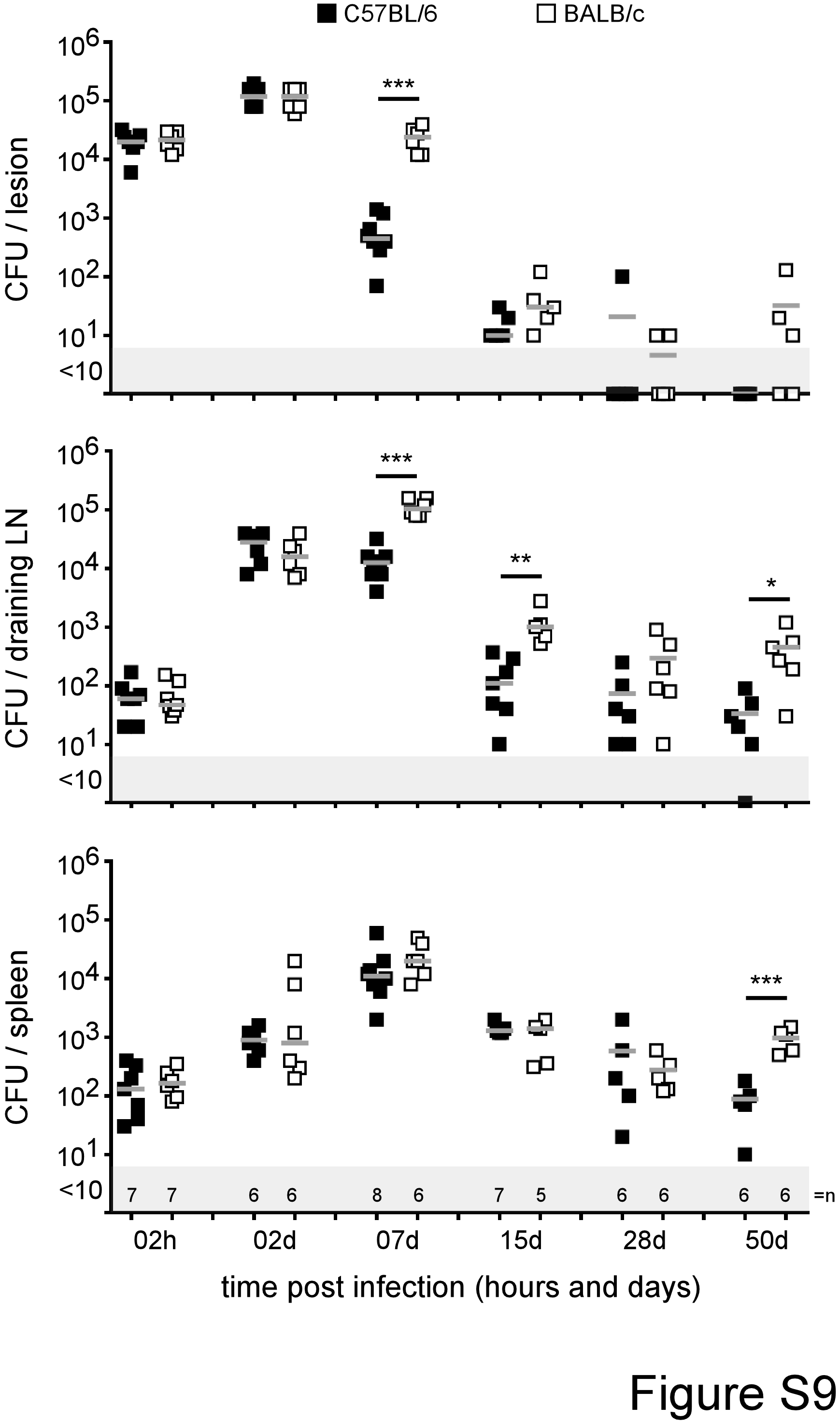

Supplement: Figure S9 — BALB/c mice are more susceptible to cutaneous Brucella infection than C57BL/6 mice. Wild-type C57BL/6 and BALB/c mice were infected intradermally with a dose of 2 × 10^4^ CFU of B. melitensis and sacrificed at the indicated times. The data represent the CFU count per organ. Gray bars represent the median. The significant differences between the indicated groups are marked with asterisks: *p < 0.1, **p < 0.01, ***p < 0.001. These results are representative of two independent experiments. LN, lymph node; d, days; n, number of mice per group. [file Image_9.TIF]

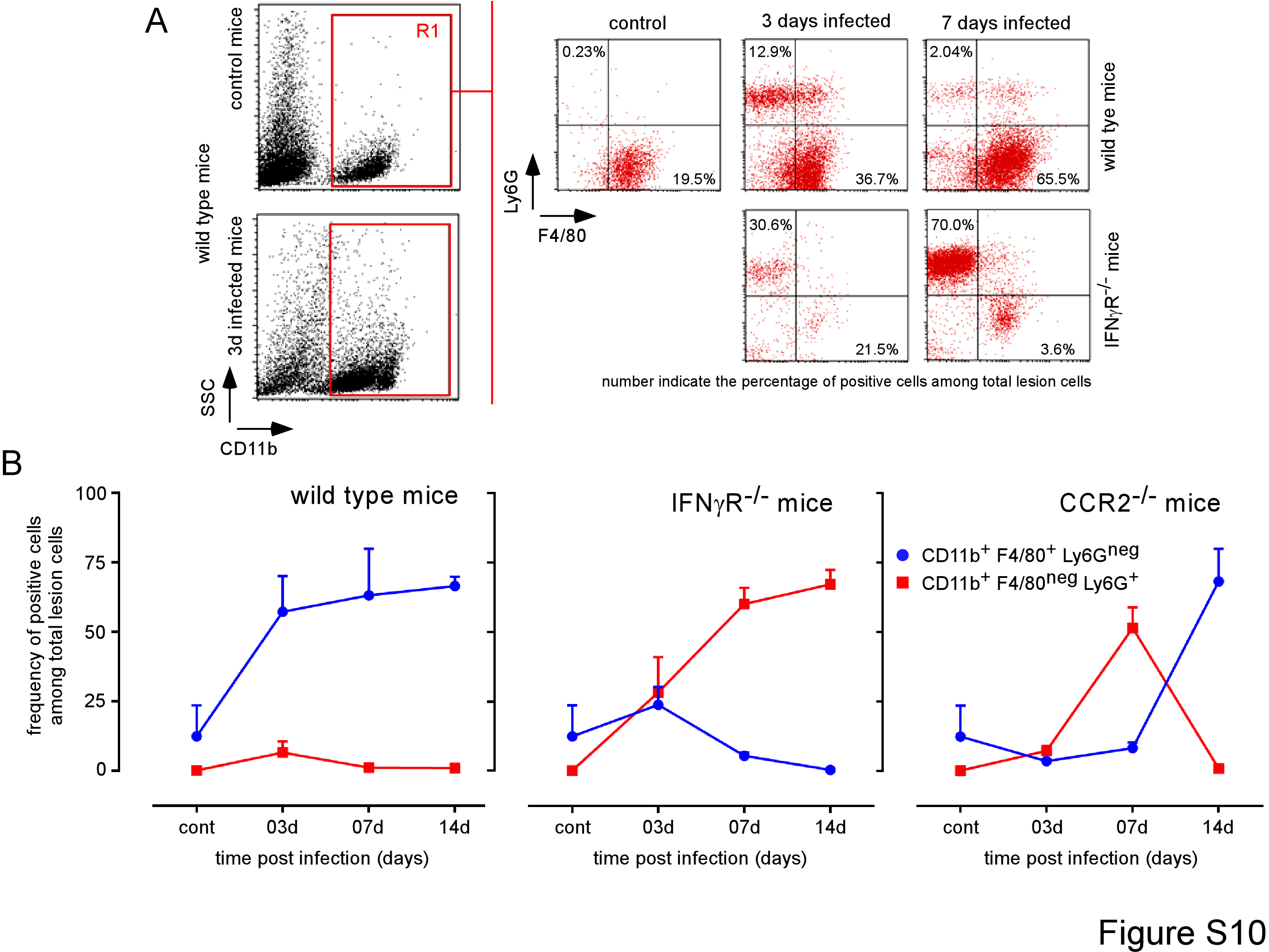

Supplement: Figure S10 — Neutrophils constitute the major population in the footpad lesion from infected IFNγR−/− mice. Wild-type, IFNγR−/−, and CCR2−/− C57BL/6 mice were infected intradermally with a dose of 2 × 10^4^ CFU of mCherry-B. melitensis. Control wild-type mice were injected with PBS. The footpad lesions were harvested at 3 and 7 days post infection and the cells were analyzed by flow cytometry. (A) Flow cytometry analysis of CD11b, F4/80, and LY6G expression on footpad cells. The data show the representative dot plot from individual mice. (B) Data represent the mean frequency (n = 4) of neutrophils and monocytes in the footpad lesion at the indicated time of infection. These results are representative of three independent experiments. Cont, control; d, days. [file Image_10.TIF]

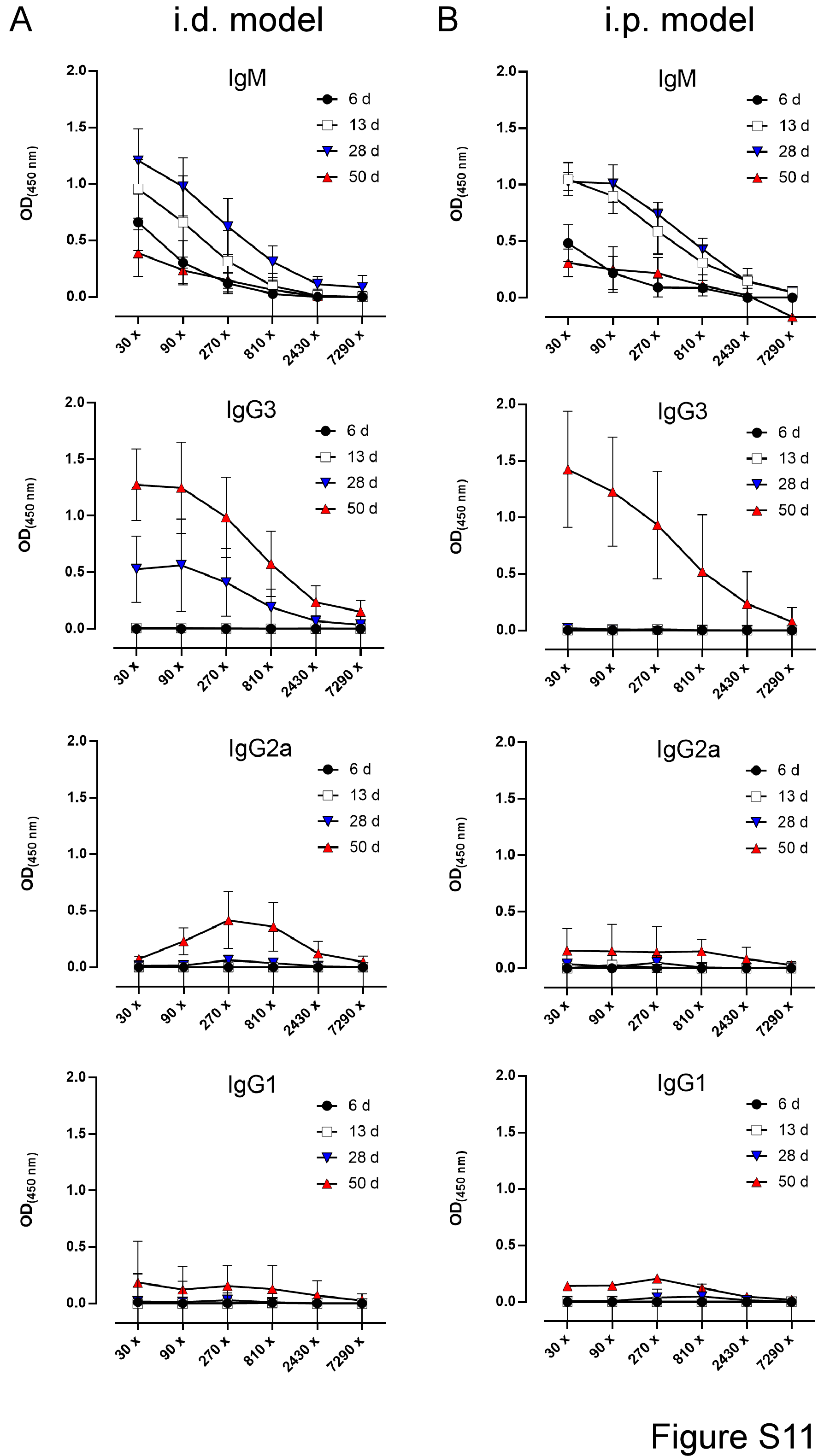

Supplement: Figure S11 — Humoral immune response induced by intradermal Brucella infection. Wild-type C57BL/6 mice were infected intradermally (A) or intraperitoneally (B) with a dose of 2 × 10^4^ CFU of mCherry-B. melitensis. Serum was collected at the indicated times, and ELISA was performed to determine the isotype distribution of the Brucella-specific antibodies. The data represent the means ± SEM of results for 8 mice. These results are representative of three independent experiments. O.D, optical density; d, days. [file Image_11.TIF]

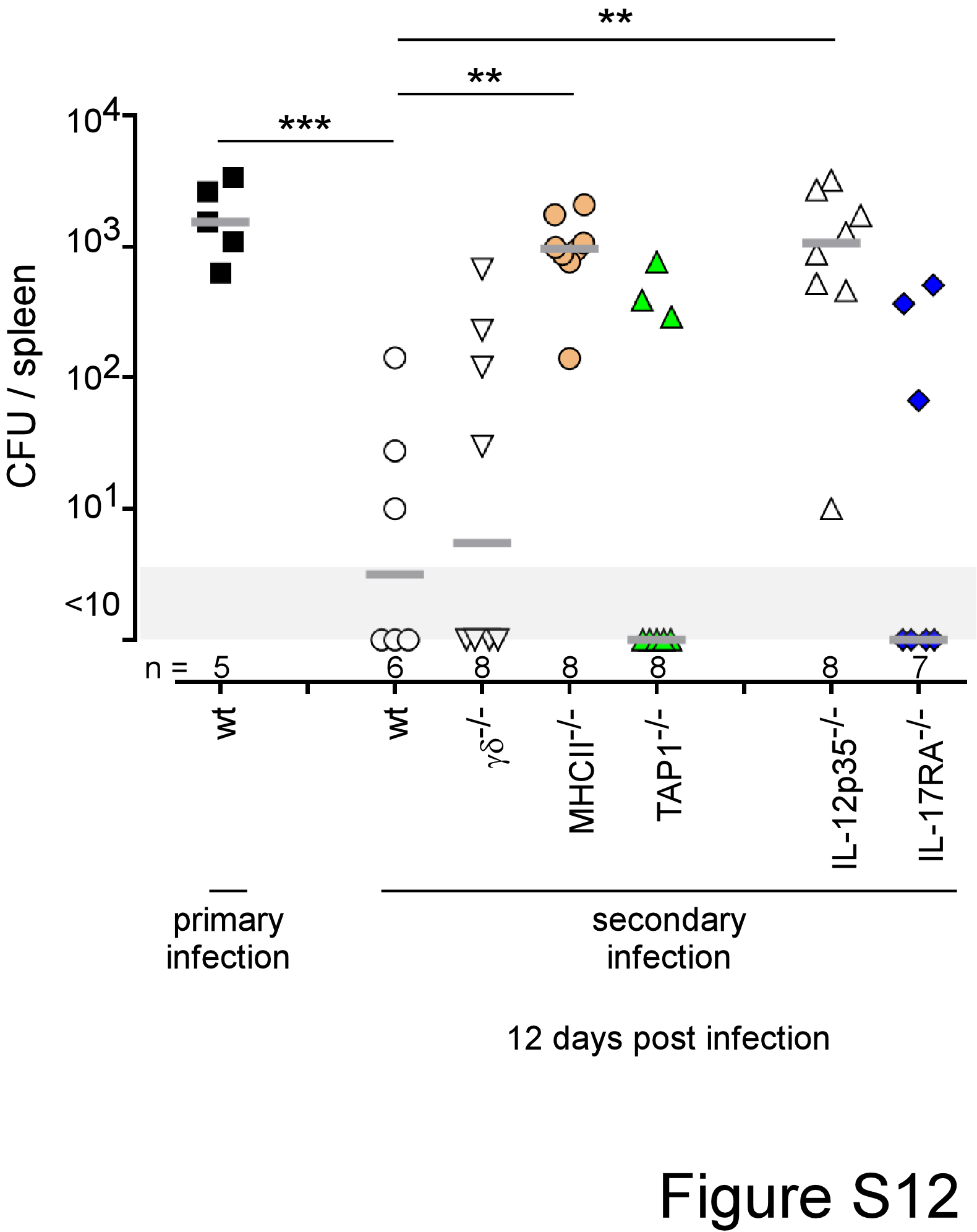

Supplement: Figure S12 — Comparison of protection in wild-type and various deficient mice previously immunized by intraperitoneal route with live B. melitensis. Wild-type, TCRγδ−/−, MHCII−/−, TAP1−/−, Il-12p35−/−, and IL-17RA−/− C57BL/6 mice were immunized i.p. with 2 × 10^4^ CFU of live wild-type B. melitensis and treated with antibiotics, as described in the Materials and Methods. Naive (primary infection group) and immunized (secondary infection group) mice were challenged i.p. with 2 × 10^4^ CFU of live mCherry-B. melitensis and sacrificed at 12 days post infection. The data represent the CFU count per spleen. Gray bars represent the median. The significant differences between the indicated groups are marked with asterisks: **p < 0.01. These results are representative of two independent experiments. n, number of mice per groups. [file Image_12.TIF]
